# Identification of candidate molecular targets of the novel antineoplastic antimitotic NP-10

**DOI:** 10.1038/s41598-019-53259-2

**Published:** 2019-11-14

**Authors:** Takuya Yokoyama, Masaki Yukuhiro, Yuka Iwasaki, Chika Tanaka, Kazunari Sankoda, Risa Fujiwara, Atsushi Shibuta, Taishi Higashi, Keiichi Motoyama, Hidetoshi Arima, Kazumasa Yoshida, Nozomi Sugimoto, Hiroyuki Morimoto, Hidetaka Kosako, Takashi Ohshima, Masatoshi Fujita

**Affiliations:** 1 Department of Cellular Biochemistry, Graduate School of Pharmaceutical Sciences, Kyushu University, 3-1-1 Maidashi, Higashiku, Fukuoka 812-8582 Japan; 2 Department of Green Pharmaceutical Chemistry, Graduate School of Pharmaceutical Sciences, Kyushu University, 3-1-1 Maidashi, Higashiku, Fukuoka 812-8582 Japan; 3 Graduate School of Pharmaceutical Sciences, Kumamoto University, 5-1 Oe-honmachi, Chuo-ku, Kumamoto 862-0973 Japan; 4 Priority Organization for Innovation and Excellence, Kumamoto University, 5-1 Oe-honmachi, Chuo-ku, Kumamoto 862-0973 Japan; 5 Laboratory of Evidence-Based Pharmacotherapy, Daiichi University of Pharmacy, 22-1 Tamagawa-cho, Minami-ku, Fukuoka 815-8511 Japan; 6 Division of Cell Signaling, Fujii Memorial Institute of Medical Sciences, Tokushima University, 3-18-15 Kuramoto-cho, Tokushima, 770-8503 Japan

**Keywords:** Biochemistry, Cancer, Chemical biology, Drug discovery, Oncology

## Abstract

We previously reported the identification of a novel antimitotic agent with carbazole and benzohydrazide structures: *N*′-[(9-ethyl-9*H*-carbazol-3-yl)methylene]-2-iodobenzohydrazide (code number NP-10). However, the mechanism(s) underlying the cancer cell-selective inhibition of mitotic progression by NP-10 remains unclear. Here, we identified NP-10-interacting proteins by affinity purification from HeLa cell lysates using NP-10-immobilized beads followed by mass spectrometry. The results showed that several mitosis-associated factors specifically bind to active NP-10, but not to an inactive NP-10 derivative. Among them, NUP155 and importin β may be involved in NP-10-mediated mitotic arrest. Because NP-10 did not show antitumor activity *in vivo* in a previous study, we synthesized 19 NP-10 derivatives to identify more effective NP-10-related compounds. HMI83-2, an NP-10-related compound with a Cl moiety, inhibited HCT116 cell tumor formation in nude mice without significant loss of body weight, suggesting that HMI83-2 is a promising lead compound for the development of novel antimitotic agents.

## Introduction

Faithful segregation of duplicated chromosomal DNA is essential to maintain genome integrity. The process is executed by the mitotic spindles composed of the spindle microtubules and centrosomes under the control of mitotic kinases such as Cdk1, Aurora kinases, and Polo-like kinases (Plks). In addition, many microtubule-associated proteins (MAPs) including the kinesin and dynein are required for faithful chromosome segregation. The mitotic spindles are among the most successful targets of anticancer chemotherapy, and they remain promising targets of novel anticancer drugs^1–4^.

Taxanes, vinca alkaloids, epothilones, and halichondrins are representative antimitotic drugs used in clinical practice, which are all tubulin-binding molecules and impede precise tubulin polymerization. While these antimitotics are effective for cancer treatment, some critical problems remain to be solved. For example, innate or acquired resistance to these agents is often observed. As microtubules also play a role in non-dividing cells including neuronal cells, these microtubule-targeting drugs perturb some important cellular processes in such cells, leading to side effects such as peripheral neuropathy. Therefore, a great deal of effort is underway to develop novel antimitotics that target non-tubulin mitotic factors such as Plks, Aurora kinases, CENP-E, and Eg5^1–4^.

We previously reported the identification of novel antimitotic agents with carbazole and benzohydrazide structures including *N*′-[(9-ethyl-9*H*-carbazol-3-yl)methylene]-2-iodobenzohydrazide (code number NP-10), *N*′-[(9-ethyl-9*H*-carbazol-3-yl)methylene]-4-iodobenzohydrazide (code number NP-14), and *N*′-[(9-ethyl-9*H*-carbazol-3-yl)methylene]-2-methylbenzohydrazide (code number HND-007). Among them, NP-14 and HND-007 inhibit tubulin polymerization *in vitro*, suggesting that mitotic arrest in cells treated with these compounds is partly due to such inhibitory activity against tubulin polymerization^5^. However, NP-10 does not suppress the tubulin polymerization, and microtubule formation is only partially inhibited in NP-10-treated cells^5^. In addition, NP-10 does not affect the kinase activity of Aurora B, Plk1, and Cdk1, or the ATPase activity of the CENP-E, Eg5, and KIFC1 kinesins^5^. The mechanism(s) underlying the cancer cell-selective inhibition of mitotic progression by NP-10 remains unclear. Here, we addressed this issue by identifying NP-10-interacting proteins by affinity purification from HeLa cell lysates using NP-10-immobilized beads followed by mass spectrometry. The results showed that several mitosis-associated factors specifically bind to active NP-10, but not to an inactive NP-10 derivative. Further analysis suggested that NUP155 and importin β are involved in NP-10-mediated mitotic arrest.

## Materials and Methods

### Cell culture

HeLa cells were obtained from JCRB Cell Bank (JCRB9004, authenticated by STR-PCR). HCT116 cells were obtained from ATCC. HEK293T cells were obtained from Dr. Masao Seto, Aichi Cancer Center Research Institute, HFF2/T (normal human fibroblasts immortalized by telomerase) cells were established in-house^5^. They were grown in Dulbecco’s modified Eagle’s medium supplemented with 8% fetal calf serum and antibiotics. HFF2/T/E7/KRAS G12V cells overexpressing the E7 of human papilloma virus type 16 and the activated KRAS mutant G12V were established previously^6^ and grown in the same medium.

### Growth inhibition assay

Growth inhibition assay was carried out essentially as described previously^5^. Briefly, cells were plated in 96-well plates at a density of 5 × 10^3^ cells/well for 24 h and then treated with the indicated compounds dissolved in DMSO for 48 h. Control cells were treated with DMSO at a final concentration of 1%. MTS/PMS solution (CellTiter Aqueous Non-Radioactive Cell proliferation Assay, #G5430, Promega) was then added, cells were further incubated for 1 h, and OD_490_ was measured to determine the number of viable cells. The percent inhibition was estimated for each drug concentration, and the IC_50_ value was calculated from the linear portion of the dose-response curve using regression analysis.

**Figure 1:**
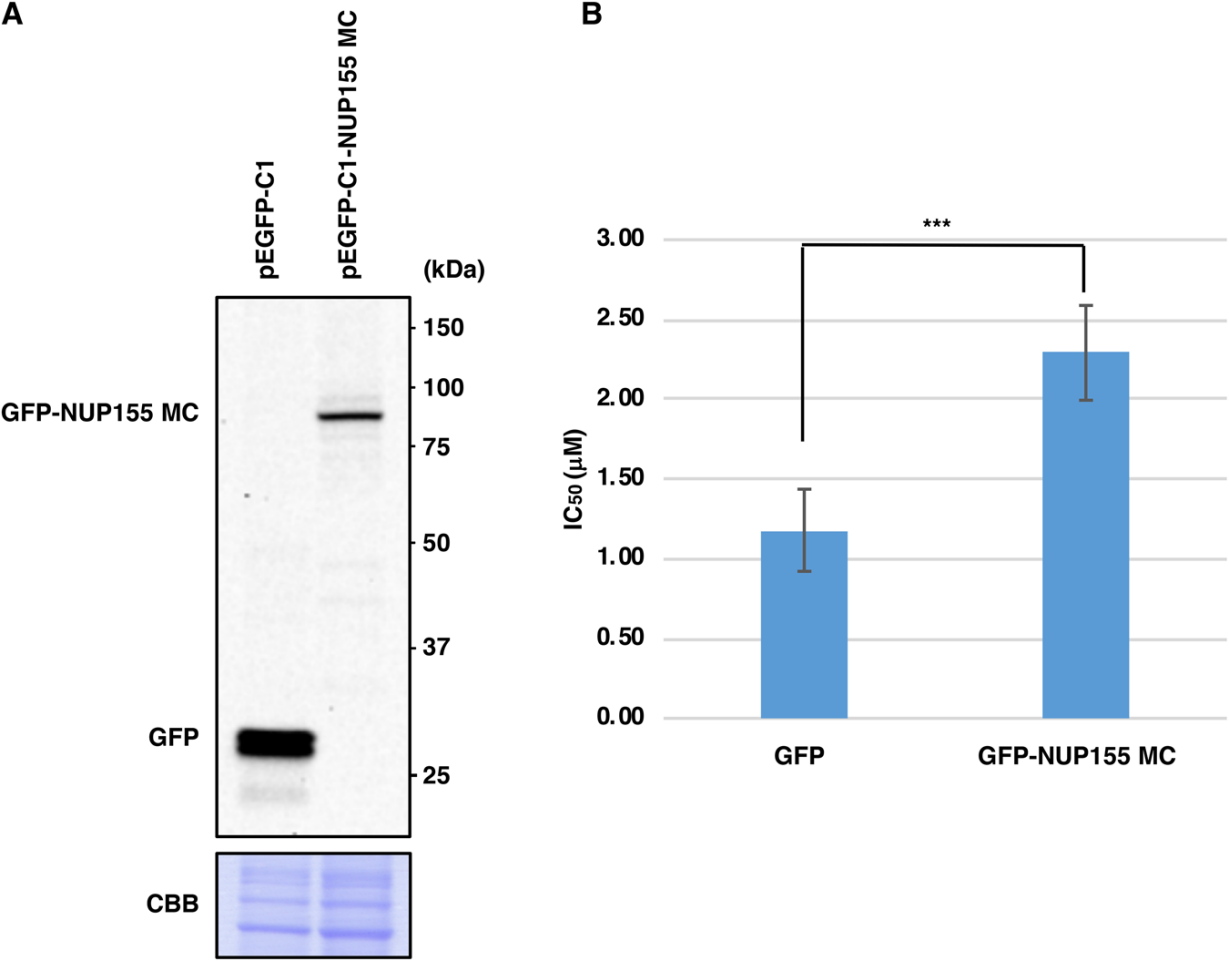
Overexpression of NUP155 MC (aa 748–1391) confers partial resistance to NP-10 cytotoxicity in HeLa cells. (**A**) HeLa cells were transfected with the indicated expression vectors for 48 h and immunoblotted with anti-GFP antibody. Proteins were stained with Coomassie Brilliant Blue (CBB) to check equal loading. (**B**) HeLa cells were transfected with the indicated expression vectors for 24 h and subjected to colony formation assays in the presence of NP-10 at various concentrations. IC_50_ values were calculated. Data represent the mean ± SD from nine independent experiments. ***p < 0.001 (two-tailed Student’s *t*-test).

**Figure 2:**
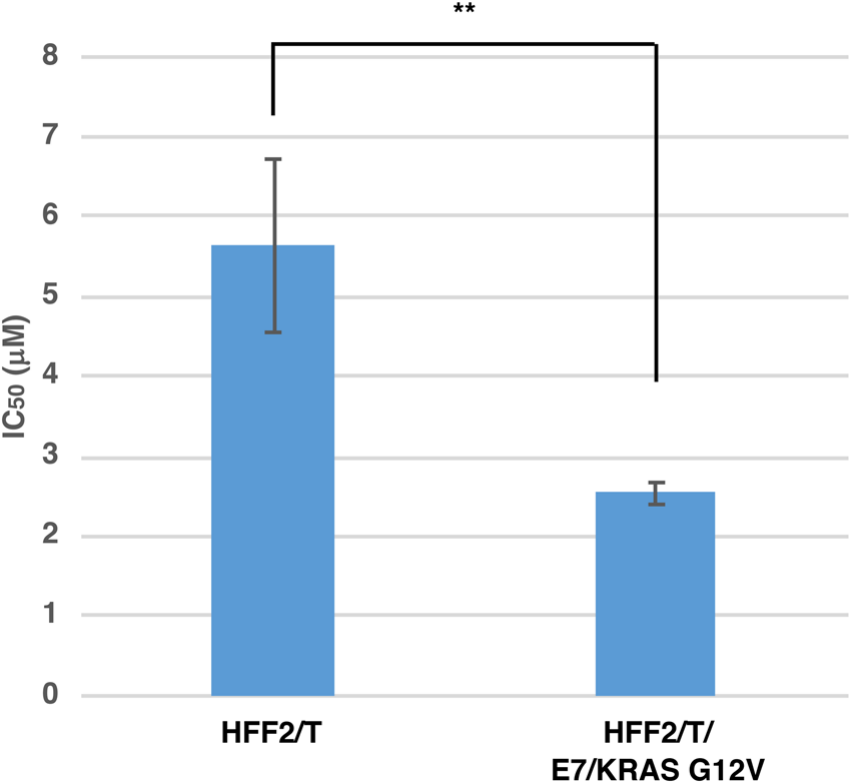
Transformed HFF2/T/E7/KRAS G12V cells are hypersensitive to NP-10 compared with parental HFF2/T cells. Parental HFF2/T and HFF2/T/E7/KRAS G12V cells were subjected to colony formation assays in the presence of NP-10 at various concentrations, and the IC_50_ values were calculated. Data represent the mean ± SD from multiple independent experiments (n = 4 for HFF2/T, n = 3 for HFF2/T/E7/KRAS G12V). **p < 0.01 (two-tailed Student’s *t*-test).

For Fig. 1B, transfected HeLa cells (see below) were replated on 12-well plates (800 cells/well) in medium containing NP-10 at various concentrations. After 7 days, colonies were stained with Giemsa solution (Wako Pure Chemical Industries). For Fig. 2, HFF2/T and HFF2/T E7/KRAS G12V cells were seeded on 12-well plates at a density of 400 cells/well in medium containing NP-10 at various concentrations. After 8 days, colonies were stained with Giemsa solution. The percentage inhibition at each drug concentration was estimated. The IC_50_ value was then calculated from the linear portion of the dose-response curve using regression analysis.

### Cell cycle analysis

Cell cycle analysis was carried out essentially as described previously^5^. Briefly, cells were treated with the indicated compounds for 24 h, and then suspended in phosphate-buffered saline (PBS) containing 0.1% TritonX-100, RNase (10 μg/mL), and propidium iodide (40 μg/mL). Cell cycle distribution was examined using a FACSCalibur or FACSVerse flow cytometer (BD Bioscience).

### Synthesis of biotinylated NP-10 and NP-14 derivatives and HMI83-2

**Figure 3:**
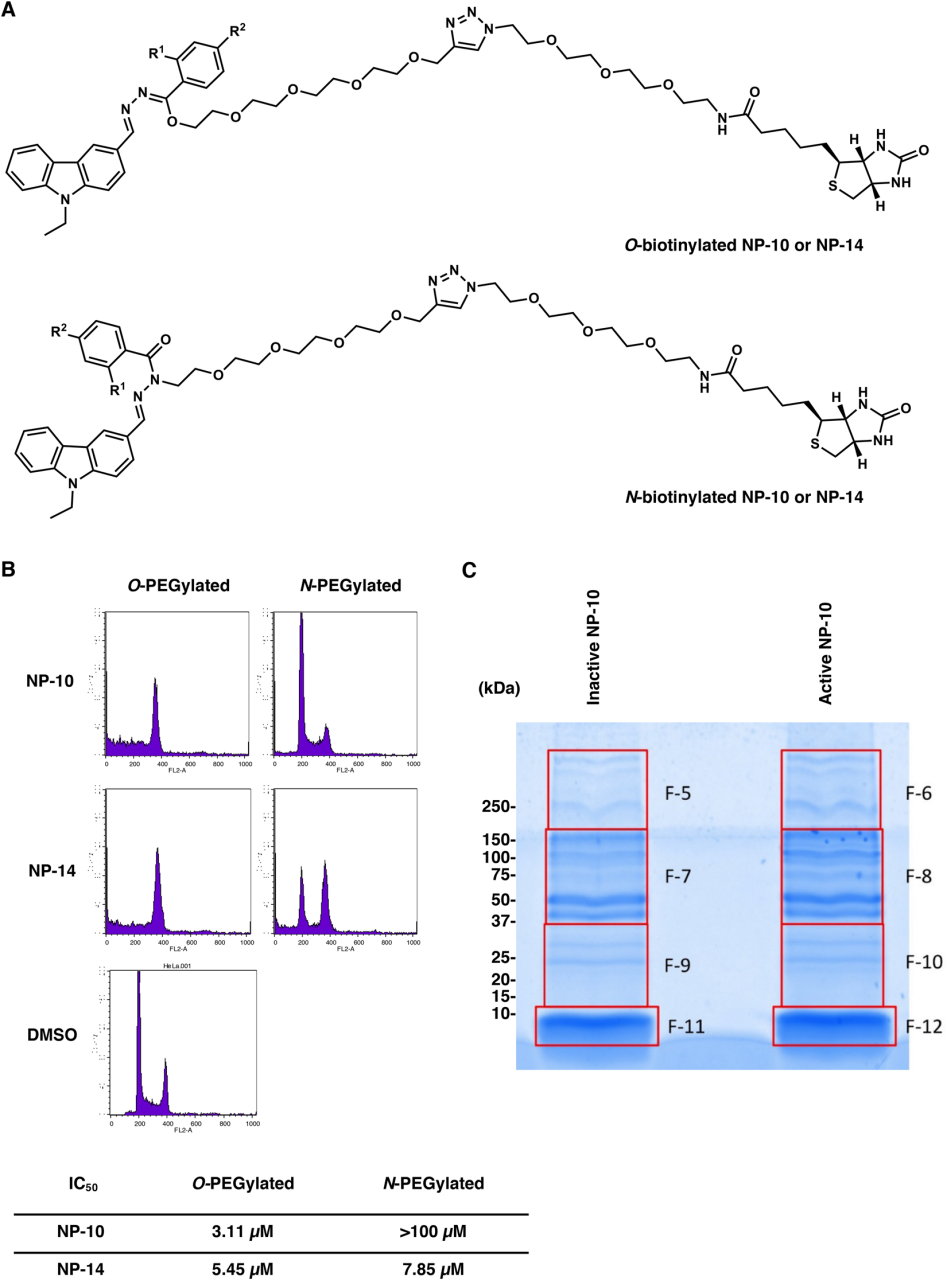
Pull-down assay with biotin-conjugated compounds. (**A**) Chemical structures of biotinylated compounds. *O*-PEGylated NP-10 (R^1^ = I, R^2^ = H) or NP-14 (R^1^ = H, R^2^ = I) linked to PEGylated biotin via the 1,2,3-triazole moiety (upper panel) and *N*-PEGylated NP-10 or NP-14 linked to PEGylated biotin via the 1,2,3-triazole moiety (lower panel) are shown. (**B**) *O*-PEGylated, but not *N*-PEGylated, NP-10 and NP-14 (without 1,2,3-triazole and PEGylated biotin) induce G2/M arrest in HeLa cells. HeLa cells were treated with vehicle (DMSO) or the indicated compounds (10 μM) for 24 h. After staining with propidium iodide, cell cycle distribution was analyzed by flow cytometry (upper panels). *In vitro* inhibition of HeLa cell growth by the compounds was also investigated (lower panel). The IC_50_ (50% inhibition concentration) values were estimated. Data represent the mean from two independent experiments. (**C**) HeLa cell lysates were incubated with the *O*-biotinylated (active) or *N*-biotinylated (inactive) NP-10 beads immobilized by biotin-avidin interaction. The pulled-down proteins were analyzed by SDS-PAGE and Coomassie Brilliant Blue (CBB) staining. The gel was cut into eight fractions as shown on the right. Proteins in each gel fraction were identified by mass spectrometry analysis.

The following compounds were synthesized for affinity purification of the binding proteins from HeLa cell lysates (Fig. 3A): 1-(1-(13-Oxo-17-((3a*S*,4*S*,6a*R*)-2-oxohexahydro-1*H*-thieno[3,4-*d*]imidazol-4-yl)-3,6,9-trioxa-12-azaheptadecyl)-1*H*-1,2,3-triazol-4-yl)-2,5,8,11-tetraoxatridecan-13-yl (*Z*)-*N*-((*E*)-(9-ethyl-9*H*-carbazol-3-yl)methylene)-2-iodobenzohydrazonate, which is *O*-PEGylated NP-10 linked to PEGylated biotin via 1,2,3-triazole moiety (code number HMI1347); *N*-(2-(2-(2-(2-(4-((*E*)-1-(9-ethyl-9*H*-carbazol-3-yl)-3-(2-iodobenzoyl)-6,9,12,15-tetraoxa-2,3-diazahexadec-1-en-16-yl)-1*H*-1,2,3-triazol-1-yl)ethoxy)ethoxy)ethoxy)ethyl)-5-((3a*S*,4*S*,6a*R*)-2-oxohexahydro-1*H*-thieno[3,4-*d*]imidazol-4-yl)pentanamide, which is *N*-PEGylated NP-10 linked to PEGylated biotin via 1,2,3-triazole moiety (code number HMI1345); 1-(1-(13-oxo-17-((3a*S*,4*S*,6a*R*)-2-oxohexahydro-1*H*-thieno[3,4-*d*]imidazol-4-yl)-3,6,9-trioxa-12-azaheptadecyl)-1*H*-1,2,3-triazol-4-yl)-2,5,8,11-tetraoxatridecan-13-yl (*Z*)-*N*-((*E*)-(9-ethyl-9*H*-carbazol-3-yl)methylene)-4-iodobenzohydrazonate, which is *O*-PEGylated NP-14 linked to PEGylated biotin via 1,2,3-triazole moiety (code number SBT171); and *N*-(2-(2-(2-(2-(4-((*E*)-1-(9-ethyl-9*H*-carbazol-3-yl)-3-(4-iodobenzoyl)-6,9,12,15-tetraoxa-2,3-diazahexadec-1-en-16-yl)-1*H*-1,2,3-triazol-1-yl)ethoxy)ethoxy)ethoxy)ethyl)-5-((3a*S*,4*S*,6a*R*)-2-oxohexahydro-1*H*-thieno[3,4-*d*]imidazol-4-yl)pentanamide, which is *N*-PEGylated NP-14 linked to PEGylated biotin via 1,2,3-triazole moiety (code number SBT-170). The synthesis procedure is described in Supplemental Information.

In addition, 19 NP-10 derivatives were synthesized including *N*′-[(9-ethyl-9*H*-carbazol-3-yl)methylene]-2-chlorobenzohydrazide (code number HMI83-2). The synthesis procedure is described in Supplemental Information.

### Identification of NP-10-binding proteins from HeLa cell lysates by pull-down assay with biotinylated compounds

The biotinylated compounds were mixed with Streptavidin Sepharose High Performance (GE Healthcare, 17-5113-01) in buffer A [50 mM Tris-HCl (pH 7.5), 50 mM KCl, 5 mM MgCl_2_, and 1 mM EDTA]. HeLa cell lysates were harvested from a 10 mL culture, resuspended in 1 mL of buffer B [buffer A containing 1 mM DTT, protease inhibitors (1 mM phenylmethylsulfonyl fluoride, 12 μg/mL aprotinin, and 1/100 protease inhibitor cocktail from Nacalai Tesque), and phosphatase inhibitors (5 mM sodium fluoride and 10 mM β-glycerophosphate)], and sonicated for 10 s eight times on ice using Bioruptor (Cosmo Bio Co., Ltd.). The soluble fraction was separated by centrifugation at 15000 rpm for 15 min and incubated with compound-immobilized beads for 4 h at 4 °C. The beads were washed three times with wash buffer (buffer A containing 0.1% NP-40 and 100 mM KCl), and bound proteins were eluted with 1 × SDS sample buffer (62.5 mM Tris-HCl pH 6.8, 2% SDS, 5% β-mercaptoethanol, 10% glycerol, and 0.01% bromophenol blue) and subjected to SDS-PAGE followed by mass spectrometry analysis or immunoblotting.

### Mass spectrometry analysis

The bound proteins were resolved by SDS-PAGE followed by Coomassie Brilliant Blue staining to visualize protein bands. The stained bands were excised and proteins in each gel slice were reduced, alkylated, and digested with trypsin (Promega) in 50 mM ammonium bicarbonate for 16 h at 37 °C. The generated peptides were then subjected to an ultra-high performance liquid chromatography system (AMR) followed by a Q Exactive mass spectrometer (Thermo Fisher Scientific). Obtained raw data were compared with the SwissProt *Homo sapiens* database using Proteome Discoverer 1.4 (Thermo Fisher Scientific) with the Mascot search engine version 2.4 (Matrix Science).

### Immunoblot analysis and antibodies

Immunoblotting was performed as described previously^7^. Antibodies used in this study were as follows: NUP155 (A303-934A, Bethyl Laboratories), IPOβ (ab2811, Abcam), KNTC1 (F2051-10H4, Sigma-Aldrich), hCAP-D2 (E4161-4C12, Sigma-Aldrich), IPO7 (ab88339, Abcam), CLTC (sc-271178, Santa Cruz), Cdc2-phosphorylated vimentin Ser55 (D076-3, MBL), GST (G7781, Sigma-Aldrich), GFP (A6455, Invitrogen), and HA-tag (clone 3F10, Roche). Secondary antibodies were HRP-rabbit anti-mouse IgG (H + L) (61-6520, Invitrogen) or HRP-goat anti-rabbit IgG (H + L) (65–6120, Invitrogen).

### siRNA experiments

siRNA oligonucleotides (IDT) with the following sequences (sense strand) were synthesized: siNUP155-1 (5′-GCAUGUCAGAUAUGGAUUAUCCUdTdT-3′), siNUP155-2 (5′-GGCAUCUACUUGUGAGUAAUGUGdGdG-3′), siIPOβ-1 (5′-GGCGGAGAUCGAAGACUAACAAAdGdC-3′), siIPOβ-2 (5′-AGAAGAGCCUAGUAAUAAUGUGAdAdG-3′), siKNTC1-1 (5′-AGCUCAUAGAUAAAGCAUGGCAGdAdA-3′), siKNTC1-2 (5′-AGAAAGGAAUGACAGUUAAGAACdCdT-3′), sihCAP-D2-1 (5′-GGCAAGGCUAUAAUAGAUGAAUUdTdG-3′), sihCAP-D2-2 (5′-GGCAGACAAGUCAGUGCUAGUAUdGdT-3′), siIPO7-1 (5′-GCCUUAGAGCUAACAAGAAGAUGdTdC-3′), siIPO7-2 (5′-AGAAGACCCUUACGAAUAUAUACdGdC-3′), control siLuci (5′-GGUUCCUGGAACAAUUGCUUUUAdCdA-3′), and control siGFP (5′-ACCCUGAAGUUCAUCUGCACCACdCdG-3′). HeLa cells (1 × 10^5^/well) seeded in 12 well plates were transfected with 18 pmol siRNA duplexes using Lipofectamine RNAiMAX (Invitrogen) for 48 h. The procedure used for co-transfection of siRNA and expression vector is described below.

### Plasmids

pGEX-6P-2-NUP155 N, pGEX-6P-1-NUP155 M, and pGEX-6P-2-NUP155 C were generated by inserting NUP155 truncated mutants (encoding amino acids [aa] 1–548, 509–992, and 960–1391, respectively) prepared from pBluescriptR-NUP155 (GE Healthcare) into pGEX-6P-1 or pGEX-6P-2 (GE Healthcare) using the In-Fusion HD cloning system (Clontech). pGEX-6P-2-IPOβ N and pGEX-6P-2-IPOβ C were similarly generated by inserting IPOβ truncated mutants (aa 1–450 and 450–876, respectively) prepared from pOTB7-IPOβ (GE Healthcare) into pGEX-6P-2.

The following primers were used: for NUP155 N, 5′-CCAGGAATTCCCGGGATGCCGTCTTCTTTGTTGGGCG-3′ (NUP155 N forward) and 5′-ATGCGGCCGCTCGAGCACAAGCCTGGTCTTCC-3′ (NUP155 N reverse); for NUP155 M, 5′-GGAATTCCCGGGTCGCTCAGCACAGGGGAGCCTTATG-3′ (NUP155 M forward) and 5′-ATGCGGCCGCTCGAGGAGACTGAGGAGCGGCC-3′ (NUP155 M reverse); for NUP155 C, 5′- CCAGGAATTCCCGGGGTTGGACTTCAGGCCTTCC-3′ (NUP155 C forward) and 5′-ATGCGGCCGCTCGAGCGGATCAGGCCCTTATGGC-3′ (NUP155 C reverse); for IPOβ N, 5′-CCAGGAATTCCCGGGATGGAGCTGATCACCATTCTCG-3′ (IPOβ N forward) and 5′-ATGCGGCCGCTCGAGAGCCAAGTAGACATCATTGATGGC-3′ (IPOβ N reverse); and for IPOβ C, 5′-CCAGGAATTCCCGGGCCCCTGCTACAGTGTCTGATTG-3′ (IPOβ C forward) and 5′-ATGCGGCCGCTCGAGTGGGGGTCCTCAGGTTATCATC-3′ (IPOβ C reverse).

pEGFP-C1-IPOβ was constructed by inserting IPOβ cDNA into pEGFP-C1 (Clontech) using the In-Fusion HD cloning system. The following primers were used: 5′-TCCGGACTCAGATCTGAGCTGATCACCATTCTCGAGAAG-3′ (IPOβ forward) and 5′-AGATCCGGTGGATCCTCAAGCTTGGTTCTTCAGTTTCCT-3′ (IPOβ reverse). pFLAG-CMV-5b-HA-IPO7 was constructed by inserting IPO7 cDNA [prepared from pCR-Blunt II-TOPO-IPO7 (ThermoFisher Scientific)] into Not I- and Sma I-digested (to remove FLAG-GRWD1) pFLAG-CMV-5b-HA-GRWD1^6^ using the In-Fusion HD cloning system. The following primers were used: 5′-CCAGACTATGCGGCCGGAGACCCCAACAACATTATCGA-3′ (IPO7 forward) and 5′-ACAGGGATGCCACCCGGGTCAATTCATCCCTGGTGCT-3′ (IPO7 reverse). pEGFP-C1-NUP155 MC was constructed by inserting NUP155 truncated mutant (aa 748–1391) into pEGFP-C1 using the In-Fusion HD cloning system. The following primers were used: 5′-TCCGGACTCAGATCTATGCGTCCTGAAAACGGAAAT-3′ (NUP155 MC forward) and 5′-AGATCCGGTGGATCCATGAAGCCGTTCTAATTTAGC-3′ (NUP155 MC reverse).

### Transfection

**Figure 4:**
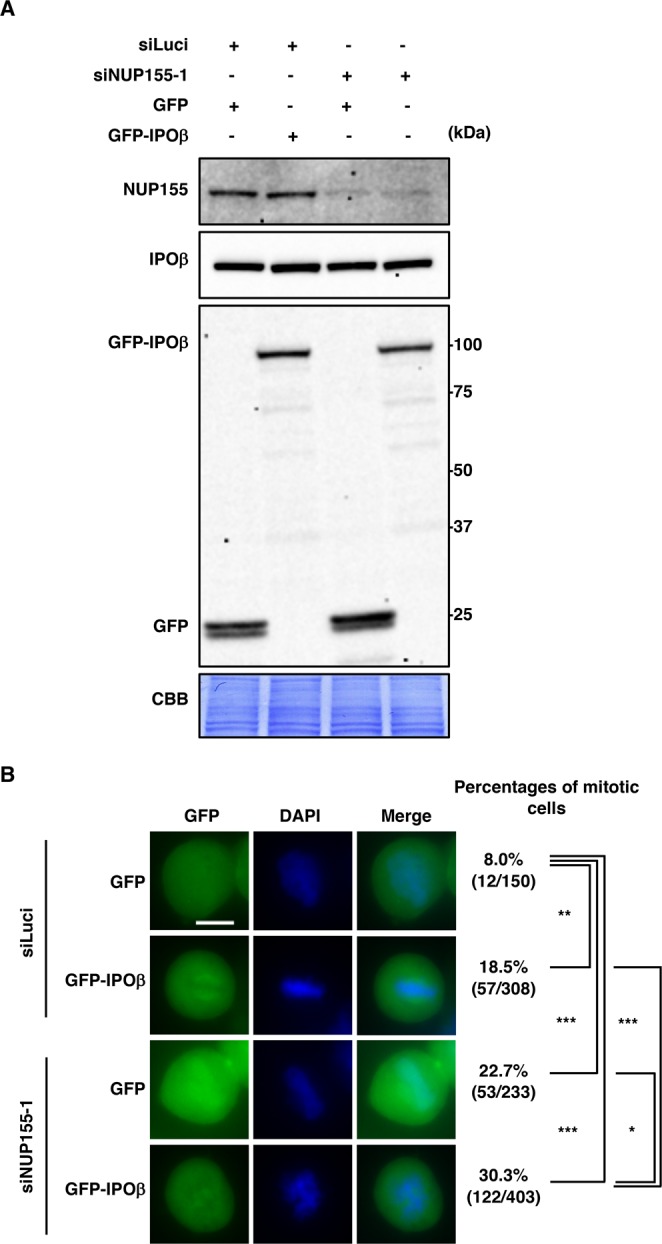
Overexpression of GFP-IPOβ and silencing of NUP155 induce mitotic arrest. (**A**) HeLa cells were co-transfected with the indicated expression vectors and siRNAs for 48 h, and immunoblotted with the indicated antibodies. (**B**) HeLa cells transfected as in (**A**) were stained with DAPI and analyzed by fluorescence microscopy. Representative images of mitotic cells are shown. Scale bar, 10 μm. Percentages of mitotic cells with GFP signals are depicted. *p < 0.05, **p < 0.01, ***p < 0.001 (χ^2^-test). Similar results were obtained in two independent experiments.

For Fig. 4, expression plasmids (0.5 μg) and siRNA (12 pmol) were transiently transfected into 1 × 10^5^ HeLa cells in 12-well plates using Lipofectamine 2000 (Invitrogen). After 16 h, cells were fed with fresh medium for immunoblotting or replated in 4-well chamber slides (LAB-TEK, Thermo Fisher) at a density of 1.67 × 10^5^ cells/well for microscopic analysis. At 48 h post-transfection, cells were used for analyses.

For Supplementary Fig. S3, expression plasmids (0.8 μg) were transiently transfected into 1 × 10^5^ HeLa and 293T cells in 12-well plates using PEImax (Polysciences). At 48 h post-transfection, cells were subjected to immunoblotting.

For Fig. 1A, expression plasmids (1.6 μg) were transiently transfected into 1 × 10^5^ HeLa cells in 12-well plates using Lipofectamine 2000 (Invitrogen). After 48 h, cells were subjected to immunoblotting.

For Fig. 1B, expression plasmids (3.0 μg) were transiently transfected into 2 × 10^5^ HeLa cells in 35 mm dishes using Lipofectamine 2000 (Invitrogen). After 24 h, cells were subjected to clonogenic assay.

### Pull-down assay of recombinant NUP155 and IPOβ with the biotinylated compounds

GST-NUP155 N, GST-NUP155 M, GST-NUP155 C, GST-IPOβ N, and IPOβ C were bacterially expressed and purified on glutathione beads as described previously^6^. Streptavidin Sepharose bound to biotinylated compounds was incubated with the purified proteins for 1 h at 4 °C. Beads were washed three times with wash buffer 1 (buffer A containing 0.02% NP-40 and 200 mM KCl) for NUP155 or wash buffer 2 (buffer A containing 0.1% NP-40 and 200 mM KCl) for IPOβ, and the bound proteins were eluted with 1 × SDS sample buffer, followed by SDS-PAGE and immunoblotting.

### Microscopic analysis

For Fig. 4B, cells were fixed with 3.7% formaldehyde in PBS for 10 min at room temperature (RT) and further treated with 100% ice-cold MeOH for 15 min. The cells were then permeabilized with 0.1% Triton X-100 in PBS for 10 min at RT and counterstained with 4,6-diamidino-2-phenylindole (DAPI). Cells were finally mounted in Fluoro-KEEPER Antifade Reagent (Nacalai Tesque) and analyzed using a KEYENCE BZ-9000 microscope.

### Tumor studies in nude mice

**Figure 5:**
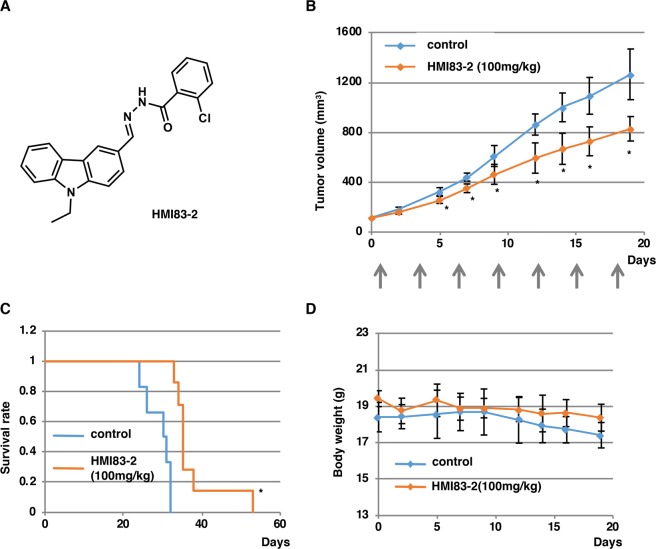
HMI83-2 suppresses the growth of HCT116 tumors in nude mice. (**A**) Chemical structure of HMI83-2. (**B**) HCT116 cells were subcutaneously inoculated into BALB/c nu/nu nude mice. The mice were treated with 100 mg/kg of HMI83-2 or control vehicle (n = 7, n = 6 respectively), as described in Materials and Methods. The mean tumor volumes (with SDs) are shown. Arrows represent the days of drug administration. *p < 0.05 (compared with vehicle; two-tailed Student’s *t*-test). (**C**) Kaplan–Meier curves obtained with the experiments shown above. *p < 0.05 (compared with vehicle; log-rank test). (**D**) Changes in body weight of the nude mice treated with HMI83-2 or control vehicle are shown.

Tumor studies in nude mice were performed essentially as described previously^5^. The protocols for animal experiments were approved by Kyushu University Animal Care and Use Committee (permit number: A23-067 and A25-023). All animal experiments were performed in accordance with the relevant national and international guidelines contained in the ‘Act on Welfare and Management of Animals’ (Ministry of Environment of Japan) and ‘Regulation of Laboratory Animals’ (Kyushu University). Four-week-old female BALB/c nu/nu nude mice were obtained from Kyushu Co., Ltd. Mice were inoculated by subcutaneous injection on the back with 4 × 10^5^ HCT116 cells mixed with Matrigel (50 μL of cell suspension + 50 μL of Matrigel, 356234, BD Biosciences). The size of the tumor was examined using digital calipers, and the volume was calculated using the following formula: tumor volume (mm^3^) = length × (width) ^2^ × $\pi$/6. Body weight and tumor size were measured three times per week. Once the tumor reached approximately 100 mm^3^, mice were randomly divided into two groups, one treated with the control vehicle mixture detailed below and the other treated with vehicle containing HMI83-2 (100 mg/kg). The indicated drug was administered intraperitoneally seven times as indicated in Fig. 5B. The vehicle mixture consisted of 40% hydroxypropyl-β-cyclodextrin (HP-β-CyD), 30% PEG400, 20% ethanol, and 10% DMSO.

### Data presentation and statistical analysis

Data presentation and statistical analysis was performed essentially as described previously^6^. Unless otherwise stated, quantitative data are shown as the mean ± SD from three or more independent experiments. For presentation of qualitative and semi-quantitative data, a representative image is displayed. For all such data, essentially the same results were observed in the multiple independent experiments. Unless otherwise stated, the statistical significance of differences between two groups was estimated by a two-tailed Student’s *t*-test. *P* values < 0.05 were considered statistically significant.

The statistical significance of the differences among more than two groups was first examined by one-way ANOVA test. Then, the difference between two groups was analyzed using the Tukey-Kramer multiple comparison test. *P* values < 0.05 were considered statistically significant.

## Results and Discussion

### Comprehensive identification of NP-10-interacting proteins

For affinity purification of NP-10-binding proteins, we prepared *O*-PEGylated or *N*-PEGylated NP-10 linked to PEGylated biotin via the 1,2,3-triazole moiety and *O*-PEGylated or *N*-PEGylated NP-14 linked to PEGylated biotin via the 1,2,3-triazole moiety (Fig. 3A and Materials and Methods). First, we determined whether these compounds had antimitotic effects comparable to those of the original NP-10 and NP-14 molecules^5^. Flow cytometry analysis showed that *O*-PEGylated NP-10 and NP-14 (without 1,2,3-triazole and PEGylated biotin), but not *N*-PEGylated NP-10 and NP-14, induced G2/M arrest and inhibited the growth of HeLa cells at concentrations comparable to those of the original compounds (Fig. 3B). Therefore, *O*-PEGylated NP-10 and NP-14 are hereafter referred to as the active forms and *N*-PEGylated NP-10 and NP-14 as the inactive forms. Biotinylated NP-10 was immobilized onto avidin-coated beads, and bound proteins were purified from HeLa cell lysates. Proteins were eluted using SDS and subjected to SDS-PAGE (Fig. 3C) followed by mass spectrometry. Among proteins identified using active NP-10 beads, those showing Mascot scores >200 and ≥2-fold higher than those of the control samples were selected (Supplementary Fig. S1).

The following proteins were selected for further analysis for the reasons listed: importin 7 because it had the highest Mascot score (2820); importin β because it showed a high Mascot score (1246) and because previous reports suggest that it is involved in regulating mitotic progression^8–11^; NUP155 because of the high Mascot score (820) and the potential involvement of nuclear pore proteins in mitotic regulation^8,12^; KNTC1 because of its high Mascot score (768) and probable involvement in mitotic regulation^13^; and hCAP-D2 because of a high Mascot score (547) and probable involvement in mitotic regulation^14,15^.

**Figure 6:**
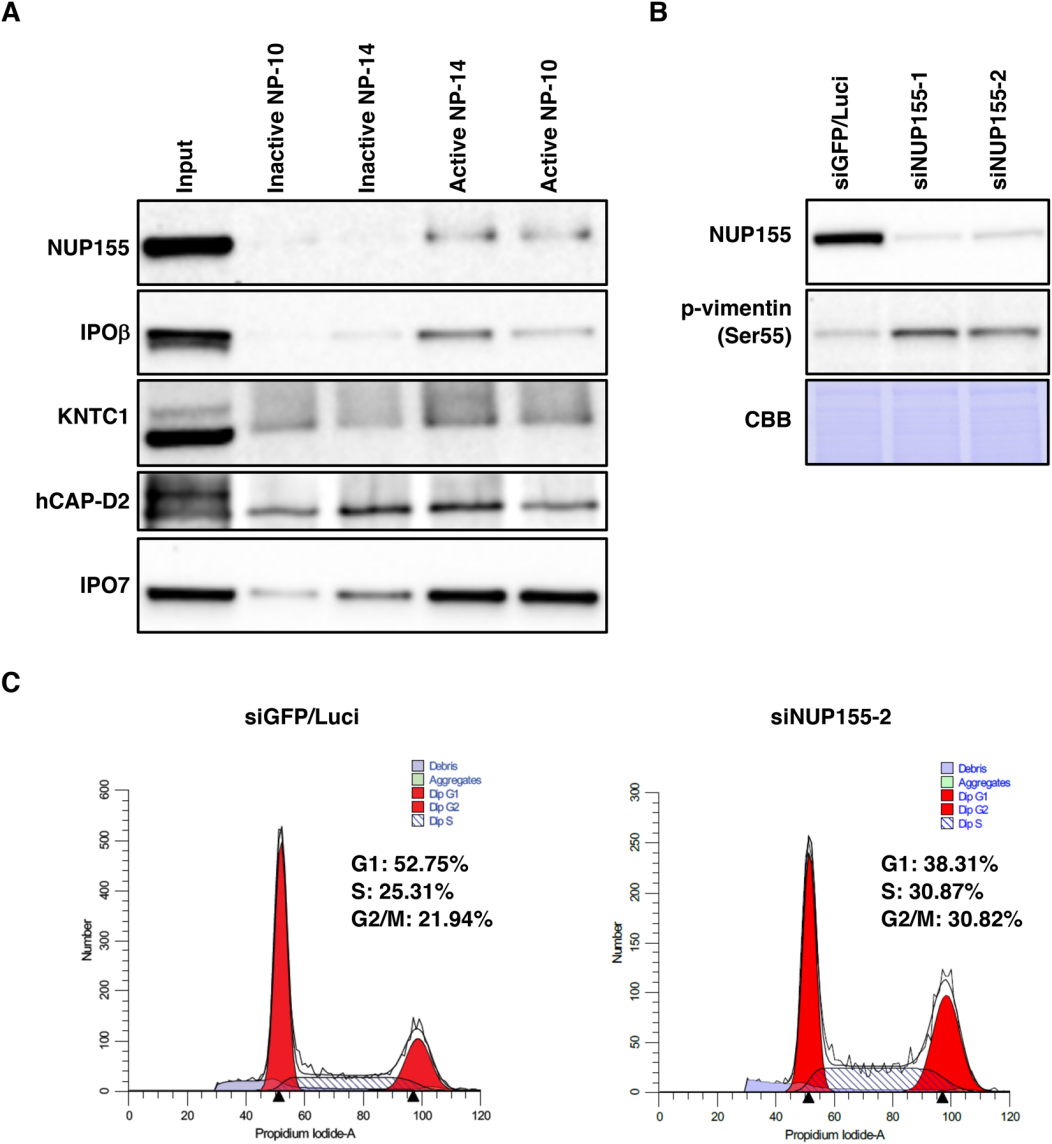
Silencing of NUP155, a potential target of NP-10, induces mitotic arrest. (**A**) HeLa cell lysates were incubated with avidin beads on which the indicated compounds were immobilized. The bound proteins and 5.4% of the input were analyzed by immunoblotting with the indicated antibodies. (**B**) HeLa cells were transfected with control (mixture of siGFP and siLuci) or NUP155-targeting (siNUP155-1 or siNUP155-2) siRNAs for 48 h. Whole cell extracts were analyzed by immunoblotting with the indicated antibodies. Phospho-vimentin (Ser55), a target of Cdk1 kinase, was used as a mitotic marker. Proteins were stained with Coomassie Brilliant Blue (CBB) to check equal loading. (**C**) HeLa cells treated as above were stained with propidium iodide and subjected to flow cytometry.

Binding of the selected proteins to active *O*-biotinylated NP-10 beads was confirmed by pull-down assay and immunoblotting. As shown in Fig. 6A, all selected proteins bound to active *O*-biotinylated NP-10 more efficiently than to inactive *N*-biotinylated NP-10. These proteins also bound to active *O*-biotinylated NP-14 (Fig. 6A). We previously showed that NP-14, but not NP-10, inhibits tubulin polymerization *in vitro*^5^. Thus, it is possible that NP-10 with an iodo moiety at the *ortho* position of the benzene ring and NP-14 with an iodo moiety at the *para* position^5^ (see also Fig. 3A) share target proteins, whereas the latter also targets tubulin.

### Silencing of NUP155, an NP-10-interacting protein, induces mitotic arrest

Because NP-10 could act as an antagonist against the identified proteins, we designed siRNAs against them and examined the effect of silencing on mitotic arrest. For this purpose, we measured the levels of phospho-vimentin Ser-55, which is phosphorylated by Cdk1, or phospho-histone H3 Ser-10, which is phosphorylated by Aurora B, as mitotic markers^5^. As shown in Figs 6B and S2, silencing of NUP155, but not the other factors including importin β and the closely related importin 7, induced mitotic arrest. The induction of mitotic arrest by NUP155 silencing was confirmed by flow cytometry (Fig. 6C).

### Overexpression of importin β, another NP-10-interaction protein, inhibits mitotic progression and the effect is enhanced by silencing NUP155

Overexpression of importin β affects the localization of certain MAPs, leading to aberrant spindle formation and delayed mitotic progression^11^. Because importin β and importin 7 efficiently bind to NP-10, we hypothesized that NP-10 may have an agonistic effect on importin β and/or importin 7. To test this hypothesis, we examined the effect of importin β and importin 7 overexpression on mitotic progression. Consistent with previous findings, overexpression of GFP-importin β (Fig. 4A) resulted in the accumulation of mitotic cells (Fig. 4B). Overexpression of importin 7 did not increase the number of mitotic cells, confirming that the effect was specific to importin β (Supplementary Fig. S3). Analysis of the effect of NUP155 silencing combined with importin β overexpression on mitotic progression showed that silencing of NUP155 additively increased the mitotic population in GFP-importin β-overexpressing HeLa cells (Fig. 4).

### Purified NUP155 and importin β directly bind to active NP-10

**Figure 7:**
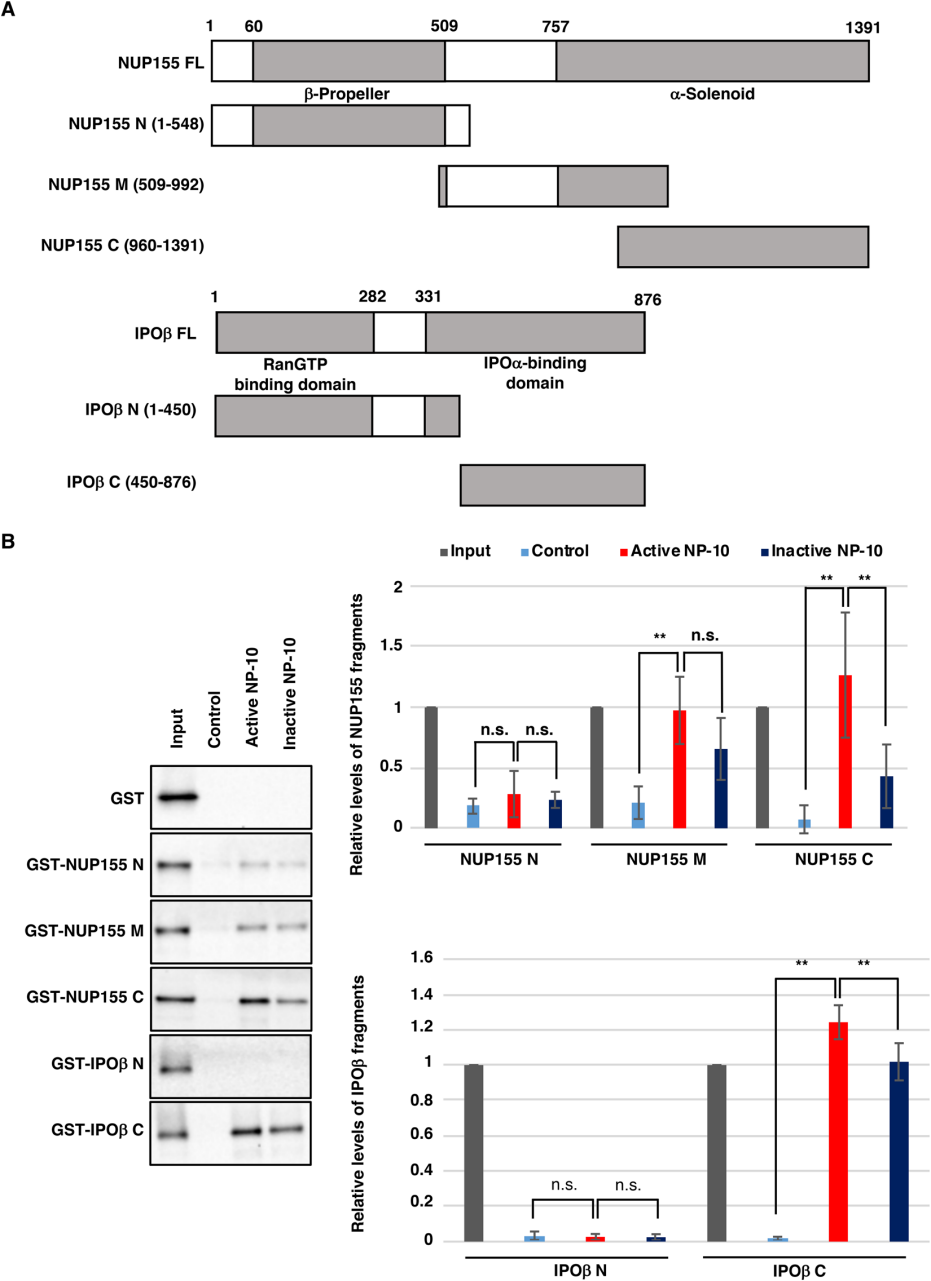
Identification of NP-10-binding domains for IPOβ and NUP155. (**A**) Schematic representation of full-length NUP155 and IPOβ (FL) and their truncated mutants. The N-terminal and C-terminal parts of NUP155 have predicted β-propeller and α-solenoid structures, respectively. The N-terminal and C-terminal parts of IPOβ are RanGTP and IPOα binding domains, respectively. (**B**) GST, GST-IPOβ mutants, or NUP155 mutants were incubated with active or inactive NP-10-immobilized beads (through biotin-avidin interaction). The pulled-down proteins, 3.5% of the input for GST-NUP155 mutants, and 5.8% of the input for GST and GST-IPOβ mutants were analyzed by immunoblotting with anti-GST antibody. Biotin-conjugated PEG linker was used as a negative control. The signal intensities of the bands were quantified and shown with the signal intensities of inputs set as 1. The mean ± SD is shown (n = 3 for NUP155 N and IPOβ N; n = 4 for NUP155 M; n = 5 for NUP155C and IPOβ C). **p < 0.01, n.s., not significant (Tukey-Kramer test).

To determine whether purified NUP155 and importin β bind to active NP-10 directly, we prepared three fragments covering full-length NUP155 (NUP155 N-terminal, mid, and C-terminal fragments; Fig. 7A) and two fragments covering full-length importin β (importin β N-terminal and C-terminal fragments; Fig. 7A), and investigated their binding to active or inactive NP-10 beads. As shown in Fig. 7B, NUP155 C-terminal fragment and importin β C-terminal fragment specifically bound to the active form of NP-10. NUP155 mid fragment showed some binding to active NP-10 beads, although to a similar extent as to inactive NP-10 beads.

### Overexpression of NUP155 confers partial resistance to NP-10 cytotoxicity in HeLa cells

To confirm that  NUP155 contributes to the effect of NP-10 on inducing mitotic arrest, we examined the effect of overexpression of NUP155 MC fragment (aa 748–1391), which includes the NP-10-binding regions, on drug sensitivity in HeLa cells. As shown in Fig. 1, overproduction of NUP155 MC decreased the cytotoxicity of NP-10 in HeLa cells. Taken together, these data supported the notion that NP-10 acts as an agonist to importin β and an antagonist to NUP155. However, it is possible that NP-10 affects other molecule(s) to induce efficient mitotic arrest in cancer cells.

### Transformed HFF2/T/E7/KRAS G12V cells are hypersensitive to NP-10 compared with parental HFF2/T cells

The molecular mechanism underlying the tumor cell-selective inhibitory effect of NP-10 remains unclear^5^. Recent studies suggest that cancer cells are under a higher replication stress than normal cells because of oncogenic hypergrowth stimuli^16,17^. Although the mechanism of the hyper-replication stress is unclear, collision between DNA replication and transcription, both of which are promoted by oncogenic stimuli, may occur more frequently in cancer cells^17^. Such collisions may stall replication forks, thereby causing hyper-replication stress. Replication stress also causes mitotic aberrations such as chromatin bridges, lagging chromosomes, and ultrafine bridges^18,19^. Therefore, we hypothesized that oncogenic hyperstimulation affects the sensitivity to NP-10. To test this hypothesis, we prepared normal human HFF2/T fibroblasts (immortalized by telomerase) and the transformed counterpart HFF2/T/E7/KRAS cells, which contain the activated KRAS mutant G12V and the E7 of human papilloma virus type 16 (HPV16)^6,20^, and investigated their sensitivity to NP-10. The results showed that transformed HFF2/T/E7/KRAS cells were hypersensitive to NP-10 (Fig. 2), which supported our hypothesis. However, other mechanism(s) may also contribute to the cancer cell selectivity of NP-10 as suggested previously^5^.

### Synthesis and evaluation of the antimitotic activity of NP-10 derivatives

**Figure 8:**
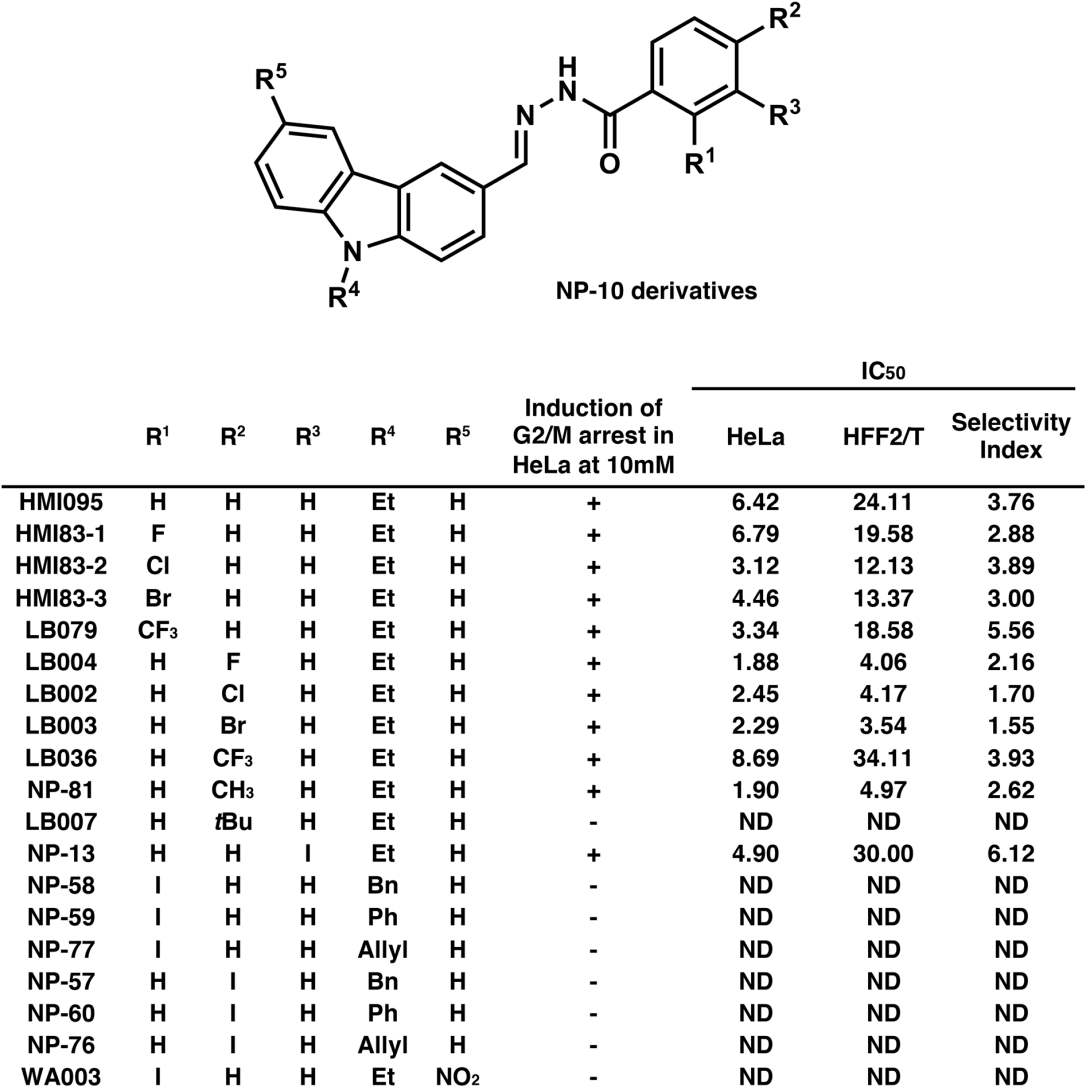
Growth inhibitory activities of various NP-10-related compounds. Chemical structures of NP-10 derivatives and their growth inhibitory activities. The IC_50_ unit was μM. The selectivity index is the ratio between the IC_50_ for HFF2/T cells and the IC_50_ for HeLa cells. ND, not determined.

In previous work, we did not observe *in vivo* antitumor activity of NP-10^5^, possibly due to poor *in vivo* stability of the iodo moiety (for detail, see a review article by Cavina *et al*.^21^) in NP-10. To obtain more effective NP-10-related compounds, we synthesized 19 NP-10 derivatives with different substituents on the benzoyl and carbazole moieties to understand the structure-activity relationship (SAR) (Fig. 8). Flow cytometric analysis was used to examine whether the compounds induced G2/M arrest in HeLa cells at 10 μM (the original NP-10 induces G2/M arrest at 10 μM^5^). Of the 19 compounds, 11 induced G2/M arrest (Fig. 8), and the SAR suggested that ethyl group on the carbazole nitrogen atom may be important for the activity. The IC_50_ of these compounds was determined by examining their effect on the growth of cervical cancer-derived HeLa cells and telomerase-immortalized normal human fibroblast HFF2/T cells (Fig. 8). In addition, we calculated the ratio between the IC_50_ for HFF2/T and the IC_50_ for HeLa as a cancer cell selectivity index^5^ (Fig. 8). The activity of the 11 compounds was comparable to that of NP-10, with IC_50_ values of 1.88–8.68 μM and selectivity indexes of 1.55–6.12 (Fig. 8). The SAR for the IC_50_ and the selectivity index suggested that the selectivity index may be better for the derivatives with substituent at the ortho position of the benzoyl moiety, whereas IC_50_ may be smaller when the substituent is present at the para position.

### HMI83-2, an NP-10-related compound with a Cl moiety, inhibits HCT116 cell tumor formation in nude mice

Based on the *in vitro* data (Fig. 8) and the fact that the I and CH_3_ moieties are generally unstable (for detail, see review articles^21–23^), we selected *N*′-[(9-ethyl-9*H*-carbazol-3-yl)methylene]-2-chlorobenzohydrazide (code number HMI83-2) (Fig. 5A) for further study. HMI83-2 shows relatively high growth inhibitory activity against HeLa cells with relatively high selectivity index and has chloro moiety instead of the iodo moiety. We examined the *in vivo* antitumor activity of HMI83-2 using a HCT116 cell xenograft model in nude mice.

Nude mice bearing HCT116 cell tumors were treated with control vehicle or HMI83-2 (100 mg/kg) seven times as indicated in Fig. 5B (intraperitoneal injection). HMI83-2 exhibited a statistically significantly higher antitumor effect than the control under these experimental conditions (Fig. 5B,C) without significant loss of body weight (Fig. 5D), suggesting that in addition to HND-007, another NP-10-related compound^5^, HMI83-2 is a promising lead compound for the development of novel antimitotic agents.

## Supplementary information

Supplementary information

## Data Availability

The datasets generated during and/or analysed during the current study and materials used in the current study are available from the corresponding author on reasonable request.
