# Supplementary material for: Identification of candidate molecular targets of the novel antineoplastic antimitotic NP-10
Source: Sci Rep. 2019 Nov 14;9:16825. doi: 10.1038/s41598-019-53259-2 (PMC6856148; doi:10.1038/s41598-019-53259-2)

Supporting Information for

Identification of candidate molecular targets of the novel antineoplastic antimitotic NP-10

Takuya Yokoyama [1, 7], Masaki Yukuhiro [1, 7], Yuka Iwasaki [1], Chika Tanaka [1], Kazunari Sankoda [1], Risa Fujiwara [2], Atsushi Shibuta [2], Taishi Higashi [3, 4], Keiichi Motoyama [3], Hidetoshi Arima [5], Kazumasa Yoshida [1], Nozomi Sugimoto [1], Hiroyuki Morimoto [2], Hidetaka Kosako [6], Takashi Ohshima [2, *], Masatoshi Fujita [1, **]

[1] Department of Cellular Biochemistry, Graduate School of Pharmaceutical Sciences, Kyushu University, 3-1-1 Maidashi, Higashiku, Fukuoka 812-8582, Japan

[2] Department of Green Pharmaceutical Chemistry, Graduate School of Pharmaceutical Sciences, Kyushu University, 3-1-1 Maidashi, Higashiku, Fukuoka 812-8582, Japan

[3] Graduate School of Pharmaceutical Sciences, Kumamoto University, 5-1 Oe-honmachi, Chuo-ku, Kumamoto 862-0973, Japan

[4] Priority Organization for Innovation and Excellence, Kumamoto University, 5-1 Oe-honmachi, Chuo-ku, Kumamoto 862-0973, Japan

[5] Laboratory of Evidence-Based Pharmacotherapy, Daiichi University of Pharmacy, 22-1 Tamagawa-cho, Minami-ku, Fukuoka 815-8511, Japan

[6] Division of Cell Signaling, Fujii Memorial Institute of Medical Sciences, Tokushima University, 3-18-15 Kuramoto-cho, Tokushima 770-8503, Japan

[7] These authors equally contributed to this work.

Table of Contents

Supplementary Materials and Methods
General procedure for the synthesis of chemical compounds
Synthesis of *O*- and *N*-PEGylated NP-10 and NP-14
Synthesis of *O*- and *N*-biotinylated NP-10 and NP-14
Synthesis of NP-10 derivatives

Supplementary References

Supplementary Figure S1. Identification of active NP-10-bound proteins.

Supplementary Figure S2. Silencing of IPOβ, KNTC1, hCAP, or IPO7, which are active NP-10-bound proteins possibly involved in mitotic regulation, does not induce mitotic arrest.

Supplementary Figure S3. Overexpression of IPO7 does not induce mitotic arrest.

Supplementary Figure S4. ^{1}H NMR analysis of compounds S4, S5, S6, S7, S8, S9, S10, S11, and HMI83-2.

Supplementary Figure S5. Source data for Figures 2, 3, 4, and 5.

Supplementary Materials and Methods

General procedure for the synthesis of chemical compounds

All synthetic reactions were performed in flame-dried or oven-dried glassware under an argon atmosphere unless otherwise noted. Reagents and catalysts were obtained from commercial sources and used as received unless otherwise stated. Solvents were purchased from commercial sources and dried over molecular sieves before use. Flash silica gel column chromatography was performed with Kanto Chemical silica gel 60N (spherical neutral, particle size: 40–50 µm).

Nuclear magnetic resonance (NMR) spectra were acquired on 500 MHz Bruker Avance III spectrometers. ^{1}H and $^{13}C\{^{1}H\}$ NMR chemical shifts are reported in ppm and referenced to tetramethylsilane or residual solvent peaks as internal standards (for $CDCl_3$, tetramethylsilane 0 ppm for ^{1}H and $CDCl_3$ 77.0 ppm for $^{13}C\{^{1}H\}$). Coupling constants are reported in hertz. The following abbreviations are used: s = singlet, d = doublet, t = triplet, q = quartet, m = multiplet, br = broad. Infrared (IR) spectra were recorded with Shimadzu IRAffinity-S1 with Quest ATR diamond accessory. High-resolution mass spectroscopy (HRMS) was performed with the Waters ACQUITY UPLC®–LCT-Premier™ XE system. Optical rotation was measured with the JASCO P2200 polarimeter.

The starting materials NP-10 and NP-14 were prepared according to the procedure published elsewhere (1). *O*-Propargyltetraethylene glycol tosylate (**S1**) (2) and 1-iodo-3,6,9,12-tetraoxapentadec-14-yne (**S2**) (3) were prepared according to the published procedure. *N*-[2-[2-[2-(2-Azidoethoxy)ethoxy]ethoxy]ethyl]biotinamide (**S3**) was purchased from the Tokyo Chemical Industry Co., Ltd. Other chemical reagents, catalysts, and solvents were obtained from commercial suppliers and used as received.

The purity of the synthesized compounds was verified by thin layer chromatography and ^{1}H NMR analysis of the products. ^{1}H NMR spectra of compounds **S4** (*O*-PEGylated NP-10), **S5** (*N*-PEGylated NP-10), **S6** (*O*-PEGylated NP-14), **S7** (*N*-PEGylated NP-14), **S8** (*O*-biotinylated NP-10), **S9** (*N*-biotinylated NP-10), **S10** (*O*-biotinylated NP-14), **S11** (*N*-biotinylated NP-14), and HMI83-2 (*N*'-[(9-ethyl-9*H*-carbazol-3-yl)methylene]-2-chlorobenzohydrazide) are shown in Supplementary Figure S4.

Synthesis of *O*- and *N*-PEGylated NP-10 and NP-14

O- and *N*-PEGylated NP-10 (**S4** and **S5**) were synthesized as follows: cesium carbonate (489 mg, 1.5 mmol, 1.5 equiv) was added to a 50 mL flask equipped with a magnetic stir bar, and the flask was flame-dried under vacuum and refilled with argon. NP-10 (467 mg, 1.0 mmol) and DMF (8.0 mL) were added to the flask, and the mixture was stirred at room temperature before addition of *O*-propargyltetraethylene glycol tosylate (**S1**) (464

mg, 1.2 mmol, 1.2 equiv) in DMF (1.0 mL + 1.0 mL for washing the addition flask). The resulting yellow suspension was stirred at room temperature for 21 h, quenched with saturated aqueous ammonium chloride and brine, and extracted with ethyl acetate. The organic layer was washed with water-brine mixture three times, dried over anhydrous sodium sulfate, filtered, and evaporated. The residue was purified by flash silica gel column chromatography using hexane/ethyl acetate = 4/1 to 0/1 as eluent to give *O*-PEGylated NP-10 (**S4**) (120 mg) as a mixture of the NP-10 and byproduct, and *N*-PEGylated NP-10 (**S5**) (502 mg) as a mixture of tosylate (**S1**). The impure *O*-PEGylated NP-10 (**S4**) was further purified by flash silica gel column chromatography using hexane/dichloromethane/ethyl acetate = 10/10/1 to 1/1/1 as eluent to give pure *O*-PEGylated NP-10 (**S4**) (61.3 mg, 9% yield) as a colorless oil. The impure *N*-PEGylated NP-10 (**S5**) was also purified by additional flash silica gel column chromatography using dichloromethane/diethyl ether = 20/1 to 2/1 as eluent to give (**S5**) (210.6 mg), which was further purified by flash silica gel column chromatography using hexane/ethyl acetate = 1/1 to 0/1 as eluent to give pure *N*-PEGylated NP-10 (**S5**) (200.0 mg, 28% yield) as a colorless film.

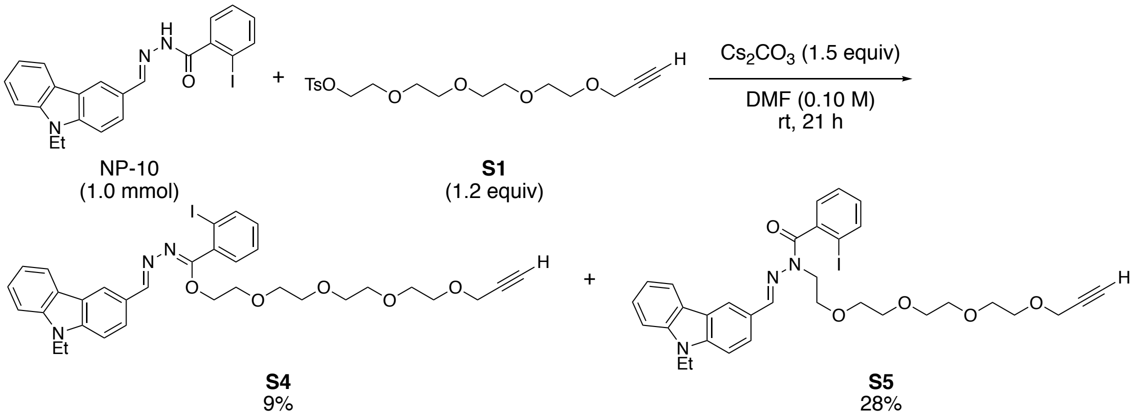

3,6,9,12-Tetraoxapentadec-14-yn-1-yl (*Z*)-*N*-((*E*)-(9-ethyl-9*H*-carbazol-3-yl)methylene)-2-iodobenzohydrazonate (S4) (*O*-PEGylated NP-10)

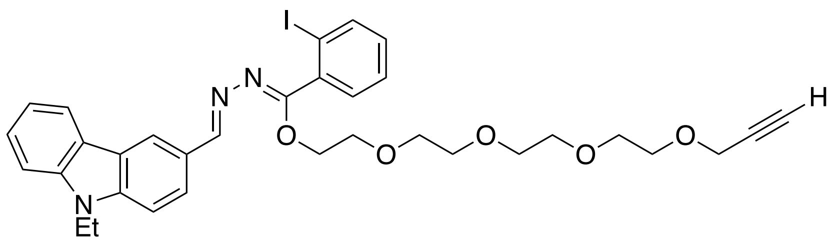

^{1}H NMR (500 MHz, $CDCl_3$) δ 8.60 (s, 1H), 8.23 (d, *J* = 1.4 Hz, 1H), 8.05 (d, *J* = 7.6 Hz, 1 H), 7.90 (dd, *J* = 0.9, 8.0 Hz, 1H), 7.73 (dd, *J* = 1.5, 8.6 Hz, 1H), 7.46 (ddd, *J* = 1.2, 7.1, 8.2 Hz, 1H), 7.42 (ddd, *J* = 1.1, 7.5, 7.5 Hz, 1H), 7.39 (d, *J* = 8.2 Hz, 1H), 7.33 (dd, *J* = 1.6, 7.6 Hz, 1H), 7.31 (d, *J* =

8.7 Hz, 1H), 7.23 (ddd, *J* = 0.8, 7.1, 7.9 Hz, 1H), 7.12 (ddd, *J* = 1.7, 7.6, 7.9 Hz, 1H), 4.62 (t, *J* = 4.9 Hz, 2H), 4.34 (q, *J* = 7.2 Hz, 2H), 4.20 (d, *J* = 2.4 Hz, 2H), 3.94 (t, *J* = 4.9 Hz, 2H), 3.78–3.72 (m, 2H), 3.72–3.58 (m, 10H), 2.42 (t, *J* = 2.4 Hz, 1H), 1.41 (t, *J* = 7.2 Hz, 3H). ^{13}C NMR (125 MHz, $CDCl_3$) δ 166.97, 159.09, 141.26, 140.30, 139.72, 138.77, 130.26, 130.07, 127.37, 126.04, 125.96, 125.51, 122.95, 122.90, 121.32, 120.58, 119.33, 108.69, 108.55, 95.47, 79.71, 74.47, 70.73, 70.67, 70.67, 70.65, 70.41, 69.23, 69.13, 66.91, 58.40, 37.67, 13.79. IR (neat) 2870, 1620, 1474, 1302, 1234, 1099, 1042, 748 cm^{-1}. HRMS (ESI-TOF) *m/z* calcd. for $C_{33}H_{37}IN_3O_5^+$ [M + H]$^+$ 682.1772, found 682.1781.

(*E*)-*N'*-((9-Ethyl-9*H*-carbazol-3-yl)methylene)-2-iodo-*N*-(3,6,9,12-tetraoxapentadec-14-yn-1-yl)benzohydrazide (S5) (*N*-PEGylated NP-10)

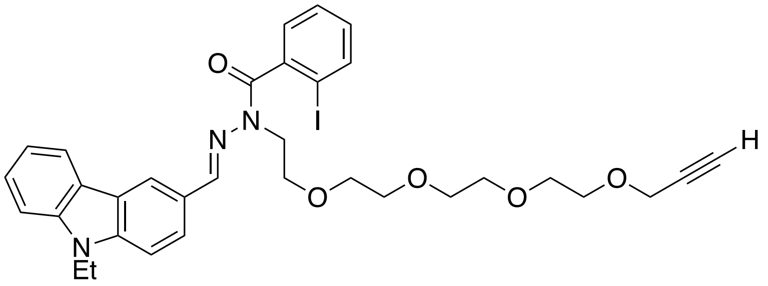

^{1}H NMR (500 MHz, $CDCl_3$) δ 8.25 (s, 1H), 8.06 (d, *J* = 1.4 Hz, 1H), 7.97 (d, *J* = 7.7 Hz, 1H), 7.90 (dd, *J* = 0.9, 8.0 Hz, 1H), 7.57–7.42 (m, 3H), 7.39 (d, *J* = 8.2 Hz, 1H), 7.34 (dd, *J* = 1.6, 7.6 Hz, 1H), 7.29 (d, *J* = 8.6 Hz, 1H), 7.24 (ddd, *J* = 0.9, 7.2, 7.9 Hz, 1H), 7.15 (ddd, *J* = 1.7, 7.8, 7.8 Hz, 1H), 4.39 (t, *J* = 5.9 Hz, 2H), 4.33 (q, *J* = 7.3 Hz, 2H), 4.14 (d, *J* = 2.4 Hz, 2H), 3.91 (t, *J* = 5.9 Hz, 2H), 3.75–3.69 (m, 2H), 3.69–3.61 (m, 6H), 3.61–3.50 (m, 4H), 2.39 (t, *J* = 2.4 Hz, 1H), 1.40 (t, *J* = 7.2 Hz, 3H). ^{13}C NMR (125 MHz, $CDCl_3$) δ 171.41, 143.71, 142.23, 140.84, 140.31, 138.33, 129.52, 127.90, 127.61, 126.04, 125.88, 124.48, 122.94, 122.84, 120.46, 120.37, 119.36, 108.74, 108.72, 93.44, 79.67, 74.46, 70.87, 70.67, 70.67, 70.59, 70.36, 69.06, 67.90, 58.35, 41.47, 37.66, 13.78. IR (neat) 2870, 1655, 1466, 1416, 1234, 1096, 746, 631 cm^{-1}. HRMS (ESI-TOF) *m/z* calcd. for $C_{33}H_{37}IN_3O_5^+$ [M + H]$^+$ 682.1772, found 682.1780.

O- and *N*-PEGylated NP-14 (**S6** and **S7**) were synthesized as follows: potassium carbonate (415 mg, 3.0 mmol, 3.0 equiv), NP-14 (467 mg, 1.0 mmol), and DMF (3.0 mL) were added to a 50 mL flask equipped with a magnetic stir bar, and the mixture was stirred at room temperature before addition of 1-iodo-3,6,9,12-tetraoxapentadec-14-yne (**S2**) (411 mg, 1.2 mmol, 1.2 equiv) in DMF (1.0 mL + 1.0 mL for washing the addition flask). The resulting yellow suspension was stirred at room temperature for 14 h and at 50°C for 5 h, quenched with saturated aqueous ammonium chloride and brine, and extracted with ethyl acetate. The organic layer was washed with water-brine mixture three times, dried

over anhydrous sodium sulfate, filtered, and evaporated. The residue was purified by flash silica gel column chromatography using hexane/ethyl acetate = 4/1 to 0/1 as eluent to give *O*-PEGylated NP-14 (**S6**) (366 mg) as a mixture of starting materials and byproduct, and *N*-PEGylated NP-14 (**S7**) (232 mg, 34% yield) as a yellow oil. The impure *O*-PEGylated NP-14 (**S6**) was further purified by flash silica gel column chromatography using hexane/dichloromethane/ethyl acetate = 10/10/1 to 1/1/2 as eluent to give pure *O*-PEGylated NP-14 (**S6**) (232 mg, 34% yield) as a yellow oil.

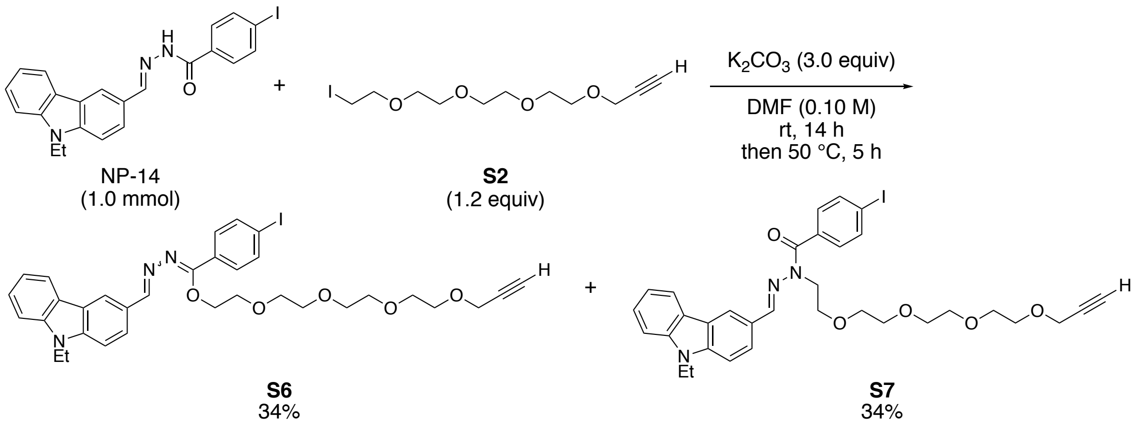

(*E*)-*N*'-((9-Ethyl-9*H*-carbazol-3-yl)methylene)-4-iodo-*N*-(3,6,9,12-tetraoxapentadec-14-yn-1-yl)benzohydrazide (S6) (*O*-PEGylated NP-14)

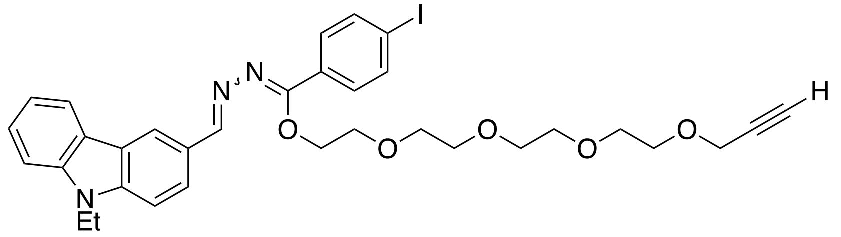

^{1}H NMR (500 MHz, $CDCl_3$) (*E*/*Z* ratio ~ 10/1; only the peaks derived from the major isomer are shown) δ 8.59 (s, 1H), 8.36 (d, *J* = 1.5 Hz, 1H), 8.12 (d, *J* = 7.7 Hz, 1H), 7.88 (dd, *J* = 1.5, 8.6 Hz, 1H), 7.84–7.76 (m, 2H), 7.69–7.60 (m, 2H), 7.49 (ddd, *J* = 1.2, 7.1, 8.2 Hz, 1H), 7.43 (d, *J* = 7.9 Hz, 1H), 7.41 (d, *J* = 8.4 Hz, 1H), 7.27 (ddd, *J* = 0.9, 7.1, 7.9 Hz, 1H), 3.95–3.89 (m, 2H), 4.39 (q, *J* = 7.3 Hz, 2H), 4.20 (d, *J* = 2.4 Hz, 2H), 3.96–3.89 (m, 2H), 3.78–3.72 (m, 2H), 3.72–3.58 (m, 10H), 2.42 (t, *J* = 2.4 Hz, 1H), 1.45 (t, *J* = 7.2 Hz, 3H). ^{13}C NMR (125 MHz, $CDCl_3$) (only the peaks derived from the major isomer are shown) δ 162.66, 158.99, 141.32, 140.37, 136.81, 132.17, 130.39, 126.11, 125.96, 125.50, 123.11, 122.93, 121.23, 120.66, 119.49, 108.76, 108.76, 97.02, 79.69, 74.49, 70.72, 70.69, 70.66, 70.65, 70.43, 69.45, 69.13, 66.38, 58.41, 37.74, 13.83. IR (neat) 2868, 1620, 1472, 1286, 1236, 1101, 1005, 808, 745, 617 cm^{-1}. HRMS (ESI-TOF) *m/z* calcd. for $C_{33}H_{37}IN_3O_5^+$ [M + H]$^+$ 682.1772, found 682.1769.

(*E*)-*N*'-((9-Ethyl-9*H*-carbazol-3-yl)methylene)-4-iodo-*N*-(3,6,9,12-tetraoxapentadec-14-yn-1-yl)benzohydrazide (S7) (*N*-PEGylated NP-14)

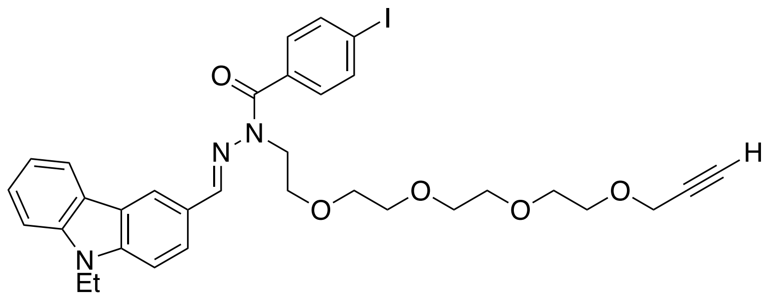

^{1}H NMR (500 MHz, $CDCl_3$) δ 8.28 (s, 1H), 8.19 (d, *J* = 1.1 Hz, 1H), 8.06 (d, *J* = 7.8 Hz, 1H), 7.88–7.74 (m, 2H), 7.67 (dd, *J* = 1.5, 8.6 Hz, 1H), 7.59–7.52 (m, 2H), 7.49 (ddd, *J* = 1.1, 7.2, 8.2 Hz, 1H), 7.42 (d, *J* = 8.2 Hz, 1H), 7.39 (d, *J* = 8.6 Hz, 1H), 7.28 (ddd, *J* = 0.8, 7.1, 7.9 Hz, 1H), 4.40 (t, *J* = 5.9 Hz, 2H), 4.37 (q, *J* = 7.3 Hz, 2H), 4.14 (d, *J* = 2.4 Hz, 2H), 3.85 (t, *J* = 5.8 Hz, 2H), 3.75–3.67 (m, 2H), 3.67–3.61 (m, 6H), 3.61–3.50 (m, 4H), 2.39 (t, *J* = 2.4 Hz, 1H), 1.44 (t, *J* = 7.2 Hz, 3H). ^{13}C NMR (125 MHz, $CDCl_3$) δ 169.96, 142.49, 140.88, 140.36, 136.48, 135.22, 131.76, 126.14, 125.84, 124.32, 123.04, 122.81, 120.75, 120.49, 119.50, 108.97, 108.80, 96.74, 79.66, 74.46, 70.85, 70.65, 70.65, 70.58, 70.36, 69.06, 68.12, 58.35, 42.06, 37.74, 13.82. IR (neat) 2866, 1643, 1584, 1416, 1236, 1101, 1063, 1007, 932, 839, 814, 745, 675, 610 cm^{-1}. HRMS (ESI-TOF) *m/z* calcd. for $C_{33}H_{37}IN_3O_5^+$ $[M + H]^+$ 682.1772, found 682.1778.

Synthesis of *O*- and *N*-biotinylated NP-10 and NP-14

The general procedure for the synthesis of *O*- and *N*-biotinylated NP-10 and NP-14 is as follows: *O*- or *N*-PEGylated NP-10 or NP-14 (0.30 mmol, 1.5 equiv), *N*-[2-[2-[2-(2-azidoethoxy)ethoxy]ethoxy]ethyl]biotinamide (**S3**) (0.20 mmol), tris(3-hydroxypropyltriazolylmethyl)amine (THPTA) (0.0020 mmol, 0.10 equiv), sodium ascorbate (0.0040 mmol, 0.20 equiv), and *tert*-butyl alcohol (0.20 mL) were added to a 4 mL Teflon-lined vial equipped with a magnetic stir bar. Copper(II) sulfate pentahydrate (0.0010 mmol, 0.050 equiv) in H_2O (0.20 mL) was added to the mixture and stirred at room temperature until the starting material (**S3**) was consumed. The crude mixture was diluted with ethyl acetate, the organic layer was washed with a mixture of brine and water, and the aqueous layer was extracted with ethyl acetate. The combined organic layer was dried over anhydrous sodium sulfate, filtered, and evaporated. The residue was purified by flash silica gel column chromatography using ethyl acetate/methanol = 4/1 to 1/2 as eluent to give *O*- or *N*-biotinylated NP-10 or NP-14.

O-biotinylated NP-10 (**S8**) was synthesized according to the general procedure

described above using *O*-PEGylated NP-10 (**S4**) (1.5 equiv) for 17 h to give *O*-biotinylated NP-10 (**S8**) (17.0 mg, 74% yield) as a colorless oil.

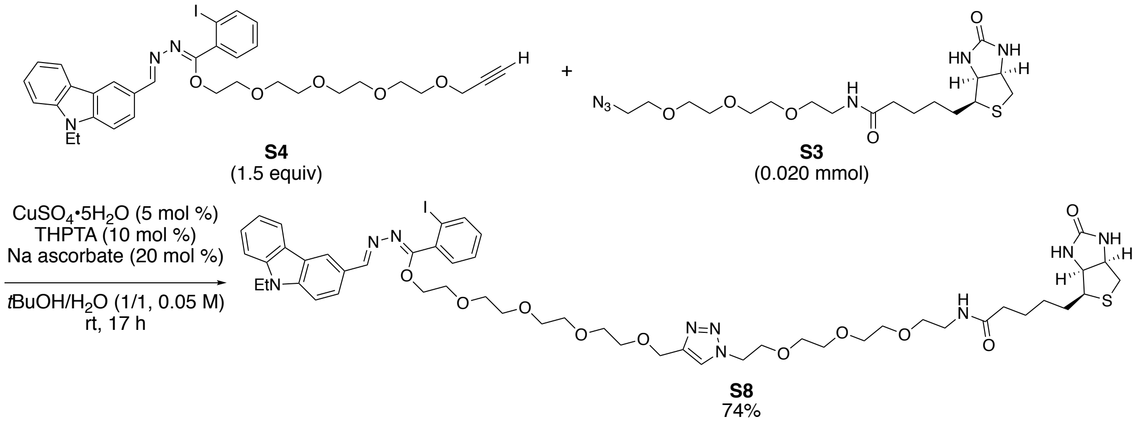

1-(1-(13-Oxo-17-((3*aS*,4*S*,6*aR*)-2-oxohexahydro-1*H*-thieno[3,4-*d*]imidazol-4-yl)-3,6,9-trioxa-12-azaheptadecyl)-1*H*-1,2,3-triazol-4-yl)-2,5,8,11-tetraoxatridecan-13-yl (*Z*)-*N*-((*E*)-(9-ethyl-9*H*-carbazol-3-yl)methylene)-2-iodobenzohydrazonate (S8) (*O*-biotinylated NP-10)

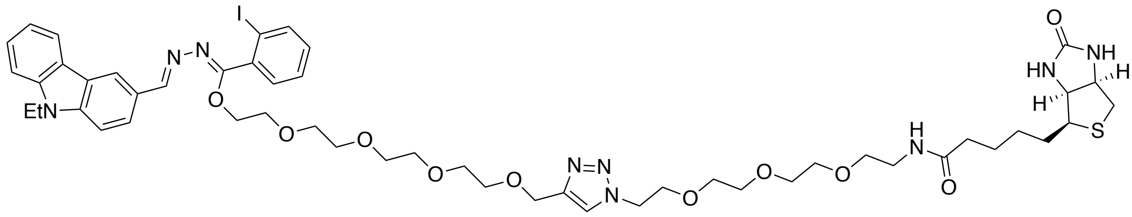

^{1}H NMR (500 MHz, $CDCl_3$) δ 8.59 (s, 1H), 8.22 (d, *J* = 1.3 Hz, 1H), 8.05 (d, *J* = 7.7 Hz, 1H), 7.89 (dd, *J* = 0.9, 8.0 Hz, 1H), 7.75 (s, 1H), 7.72 (dd, *J* = 1.5, 8.6 Hz, 1H), 7.46 (ddd, *J* = 1.1, 7.2, 8.2 Hz, 1H), 7.42 (ddd, *J* = 1.1, 7.6, 7.6 Hz, 1H), 7.39 (d, *J* = 8.2 Hz, 1H), 7.32 (dd, *J* = 1.7, 7.6 Hz, 1H), 7.31 (d, *J* = 8.5 Hz, 1H), 7.23 (ddd, *J* = 0.8, 7.1, 7.9 Hz, 1H), 7.12 (ddd, *J* = 1.8, 7.9, 7.9 Hz, 1H), 6.62 (t, *J* = 5.5 Hz, 1H), 6.21 (brs, 1H), 5.31 (brs, 1H), 4.67 (s, 2H), 4.61 (t, *J* = 4.9 Hz, 2H), 4.52 (t, *J* = 5.1 Hz, 2H), 4.45 (dd, *J* = 5.0, 7.7 Hz, 1H), 4.34 (q, *J* = 7.2 Hz, 2H), 4.28 (dd, *J* = 5.0, 6.5 Hz, 1H), 3.93 (t, *J* = 5.0 Hz, 2H), 3.86 (t, *J* = 5.3 Hz, 2H), 3.79–3.71 (m, 2H), 3.71–3.61 (m, 10H), 3.61–3.56 (m, 8H), 3.54 (t, *J* = 5.1 Hz, 2H), 3.48–3.36 (m, 2H), 3.11 (dt, *J* = 4.7, 7.4 Hz, 1H), 2.87 (dd, *J* = 5.0, 12.8 Hz, 1H), 2.71 (d, *J* = 12.8 Hz, 1H), 2.18 (t, *J* = 7.2 Hz, 2H), 1.80–1.56 (m, 4H), 1.43 (tt, *J* = 7.7, 7.7 Hz, 2H), 1.41 (t, *J* = 7.2 Hz, 3H). ^{13}C NMR (125 MHz, $CDCl_3$) δ 173.17, 166.91, 163.63, 159.09, 144.93, 141.23, 140.27, 139.66, 138.74, 130.28, 130.05, 127.37, 125.98, 125.97, 125.48, 123.84, 122.90, 122.87, 121.28, 120.55, 119.33, 108.69, 108.55, 95.44, 70.67, 70.60, 70.60, 70.55, 70.50, 70.47, 70.36, 70.33, 70.05, 69.89, 69.68, 69.42, 69.19, 66.87, 64.59, 61.71, 60.09, 55.42, 50.14, 40.47, 39.11, 37.65, 35.83, 28.11,

28.06, 25.50, 13.77. IR (neat) 3291, 2868, 2361, 1699, 1622, 1472, 1304, 1123, 748 cm^{-1}. HRMS (ESI-TOF) *m/z* calcd. for $C_{51}H_{69}IN_9O_{10}S^+$ $[M + H]^+$ 1126.3927, found 1126.3942. $[\alpha]^{25}_D$ +12.3 (*c* 0.86, $CHCl_3$).

N-biotinylated NP-10 (**S9**) was synthesized according to the general procedure described above using *N*-PEGylated NP-10 (**S5**) (1.6 equiv) for 24 h to give *N*-biotinylated NP-10 (**S9**) (19.5 mg, 84% yield) as a colorless oil.

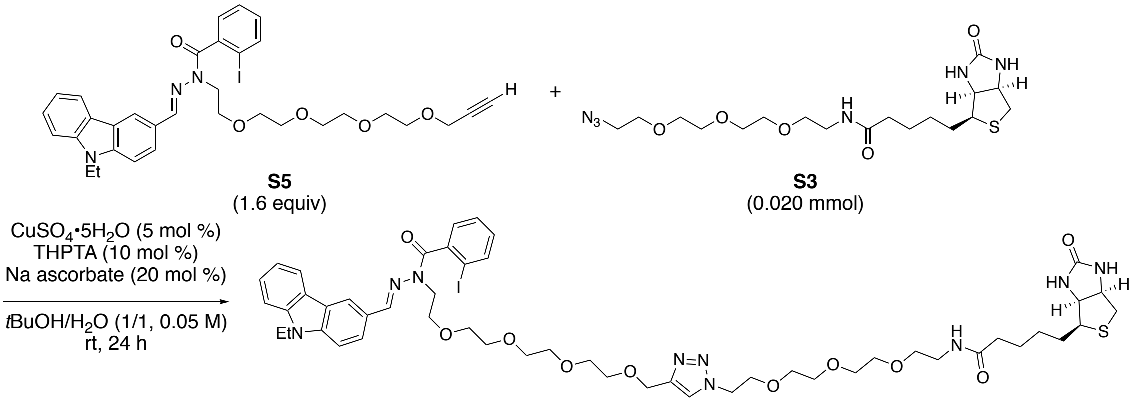

S9
84%

_N_-(2-(2-(2-(2-(4-((_E_)-1-(9-Ethyl-9_H_-carbazol-3-yl)-3-(2-iodobenzoyl)-6,9,12,15-tetraoxa-2,3-diazahexadec-1-en-16-yl)-1_H_-1,2,3-triazol-1-yl)ethoxy)ethoxy)ethoxy)ethyl)-5-((3_aS_,4_S_,6_aR_)-2-oxohexahydro-1_H_-thieno[3,4-_d_]imidazol-4-yl)pentanamide (S9) (_N_-biotinylated NP-10)

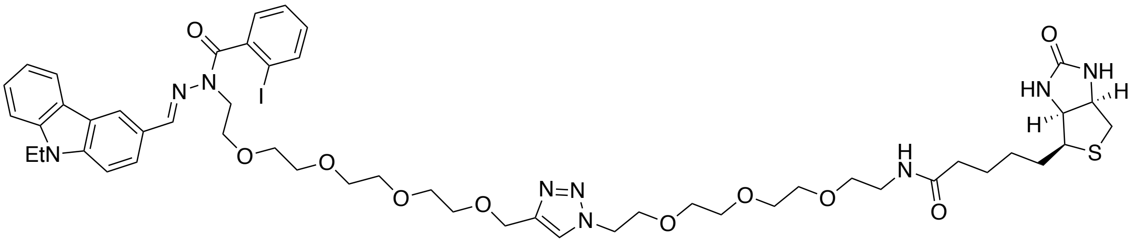

^{1}H NMR (500 MHz, $CDCl_3$) δ 8.24 (s, 1H), 8.05 (d, *J* = 1.3 Hz, 1H), 7.96 (d, *J* = 7.7 Hz, 1H), 7.90 (dd, *J* = 0.7, 8.0 Hz, 1H), 7.72 (s, 1H), 7.55–7.42 (m, 3H), 7.39 (d, *J* = 8.2 Hz, 1H), 7.34 (dd, *J* = 1.6, 7.6 Hz, 1H), 7.28 (d, *J* = 8.6 Hz, 1H), 7.24 (ddd, *J* = 0.6, 7.1, 7.8 Hz, 1H), 7.15 (ddd, *J* = 1.7, 7.8, 7.8 Hz, 1H), 6.63 (t, *J* = 5.1 Hz, 1H), 6.24 (brs, 1H), 5.36 (brs, 1H), 4.63 (s, 2H), 4.51 (t, *J* = 5.1 Hz, 2H), 4.45 (dd, *J* = 5.1, 7.6 Hz, 1H), 4.39 (t, *J* = 5.8 Hz, 2H), 4.33 (q, *J* = 7.2 Hz, 2H), 4.27 (dd, *J* = 5.1, 7.0 Hz, 1H), 3.90 (t, *J* = 5.9 Hz, 2H), 3.85 (t, *J* = 5.3 Hz, 2H), 3.75–3.68 (m, 2H), 3.68–3.61 (m, 6H), 3.61–3.55 (m, 12H), 3.54 (t, *J* = 5.3 Hz, 2H), 3.47–3.34 (m, 2H), 3.11 (dt, *J* = 4.8, 7.4 Hz, 1H), 2.86

(dd, *J* = 5.0, 12.8 Hz, 1H), 2.71 (d, *J* = 12.8 Hz, 1H), 2.18 (t, *J* = 7.4 Hz, 2H), 1.80–1.57 (m, 4H), 1.41 (tt, *J* = 7.4, 7.4 Hz, 2H), 1.40 (t, *J* = 7.2 Hz, 3H). ^{13}C NMR (125 MHz, $CDCl_3$) δ 173.16, 171.39, 163.68, 144.86, 143.66, 142.18, 140.81, 140.28, 138.30, 129.53, 127.87, 127.61, 126.04, 125.82, 124.46, 123.80, 122.89, 122.78, 120.43, 120.33, 119.35, 108.75, 108.73, 93.41, 70.80, 70.62, 70.62, 70.49, 70.48, 70.43, 70.35, 70.31, 70.03, 69.88, 69.62, 69.40, 67.74, 64.53, 61.71, 60.10, 55.42, 50.11, 41.37, 40.46, 39.09, 37.65, 35.82, 28.11, 28.05, 25.49, 13.76. IR (neat) 3298, 2866, 1701, 1654, 1466, 1418, 1234, 1099, 731 cm^{-1}. HRMS (ESI-TOF) *m/z* calcd. for $C_{51}H_{69}IN_9O_{10}S^+$ [M + H]$^+$ 1126.3927, found 1126.3927. $[\alpha]^{27}_D$ +12.9 (*c* 1.10, $CHCl_3$).

O-biotinylated NP-14 (**S10**) was synthesized according to the general procedure described above using *O*-PEGylated NP-14 (**S6**) (1.5 equiv) with additional methanol (0.20 mL) as solvent for 17 h to give *O*-biotinylated NP-10 (**S10**) (19.0 mg, 83% yield) as a colorless oil.

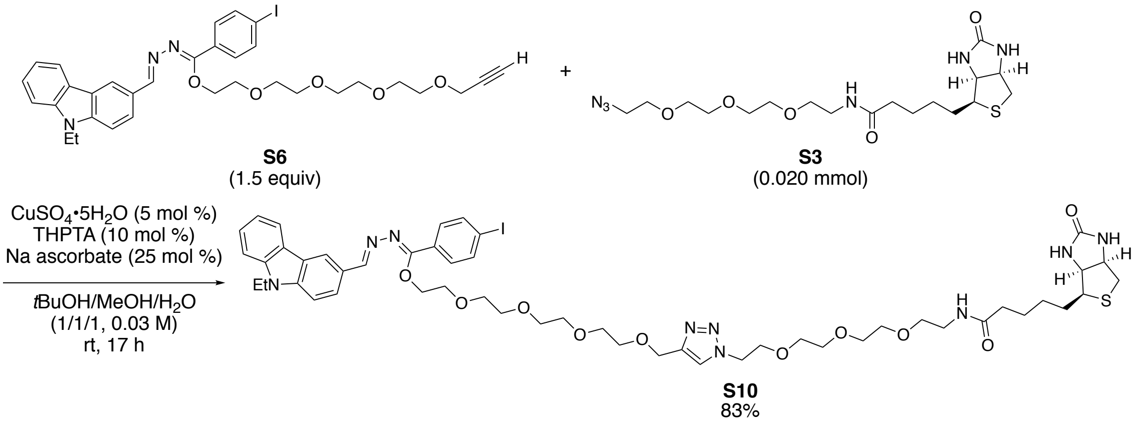

1-(1-(13-Oxo-17-((3*aS*,4*S*,6*aR*)-2-oxohexahydro-1*H*-thieno[3,4-*d*]imidazol-4-yl)-3,6,9-trioxa-12-azaheptadecyl)-1*H*-1,2,3-triazol-4-yl)-2,5,8,11-tetraoxatridecan-13-yl (*Z*)-*N*-((*E*)-(9-ethyl-9*H*-carbazol-3-yl)methylene)-4-iodobenzohydrazonate (S10) (*O*-biotinylated NP-14)

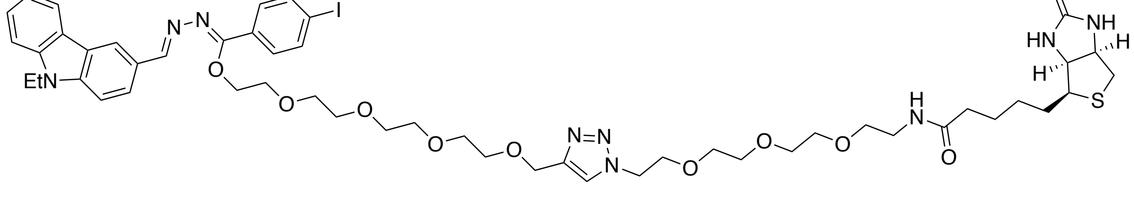

^{1}H NMR (500 MHz, $CDCl_3$) δ 8.58 (s, 1H), 8.35 (d, *J* = 1.4 Hz, 1H), 8.11 (d, *J* = 7.7 Hz, 1H), 7.88 (dd, *J* = 1.5, 8.6 Hz, 1H), 7.83–7.77 (m, 2H), 7.75 (s, 1H), 7.69–7.59 (m, 2H), 7.49 (ddd, *J* = 1.1, 7.2, 8.2 Hz, 1H), 7.42 (d, *J* = 7.3 Hz, 1H), 7.41 (d, *J* = 8.4 Hz,

1H), 7.27 (ddd, *J* = 0.8, 7.1, 7.9 Hz, 1H), 6.63 (t, *J* = 5.3 Hz, 1H), 6.25 (brs, 1H), 5.34 (brs, 1H), 4.68 (s, 2H), 4.53 (t, *J* = 5.8 Hz, 2H), 4.52 (t, *J* = 5.6 Hz, 2H), 4.46 (dd, *J* = 5.0, 7.7 Hz, 1H), 4.38 (q, *J* = 7.3 Hz, 2H), 4.28 (ddd, *J* = 0.9, 4.6, 5.9 Hz, 1H), 3.90 (t, *J* = 4.8 Hz, 2H), 3.86 (t, *J* = 5.3 Hz, 2H), 3.78–3.71 (m, 2H), 3.71–3.62 (m, 10H), 3.62–3.56 (m, 8H), 3.54 (t, *J* = 5.3 Hz, 2H), 3.48–3.35 (m, 2H), 3.11 (dt, *J* = 4.7, 7.4 Hz, 1H), 2.87 (dd, *J* = 5.0, 12.8 Hz, 1H), 2.71 (d, *J* = 12.8 Hz, 1H), 2.18 (t, *J* = 7.3 Hz, 2H), 1.82–1.55 (m, 4H), 1.45 (t, *J* = 7.2 Hz, 3H), 1.42 (tt, *J* = 7.9, 7.9 Hz, 2H). ^{13}C NMR (125 MHz, $CDCl_3$) δ 173.17, 163.67, 162.59, 159.00, 144.91, 141.30, 140.34, 136.80, 132.14, 130.35, 126.11, 125.90, 125.47, 123.82, 123.07, 122.88, 121.20, 120.63, 119.48, 108.76, 108.76, 97.02, 70.67, 70.62, 70.60, 70.54, 70.49, 70.49, 70.36, 70.33, 70.04, 69.90, 69.68, 69.42, 69.40, 66.35, 64.58, 61.72, 60.10, 55.44, 50.14, 40.47, 39.11, 37.72, 35.84, 28.12, 28.06, 25.50, 13.81. IR (neat) 2868, 1697, 1614, 1474, 1234, 1096, 1007, 731 cm^{-1}. HRMS (ESI-TOF) *m/z* calcd. for $C_{51}H_{69}IN_9O_{10}S^+$ [M + H]$^+$ 1126.3927, found 1126.3921. $[\alpha]^{25}_D$ +13.1 (*c* 0.84, $CHCl_3$).

N-biotinylated NP-14 (**S11**) was synthesized according to the general procedure described above using *N*-PEGylated NP-14 (**S7**) (1.5 equiv) with additional methanol (0.20 mL) as solvent for 24 h to give *N*-biotinylated NP-14 (**S11**) (19.3 mg, 84% yield) as a colorless oil.

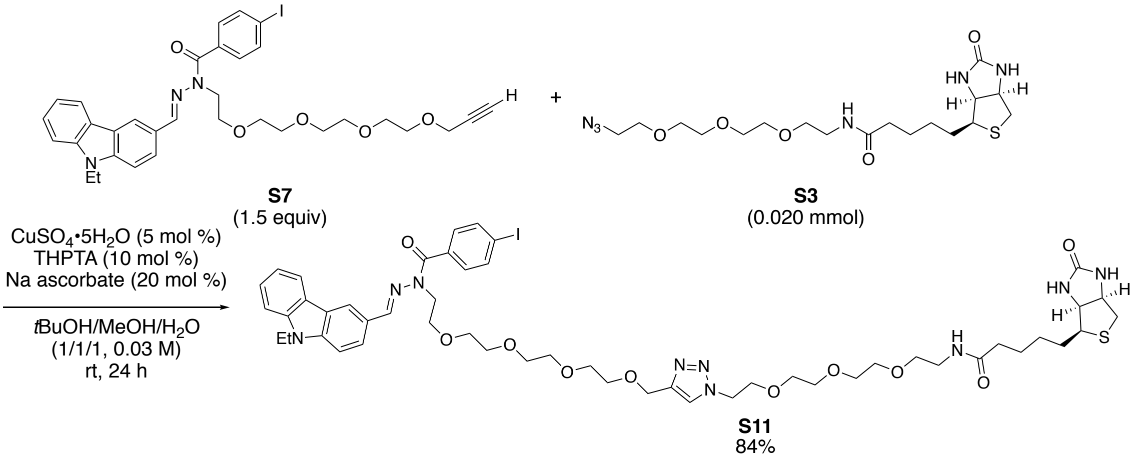

***N*-(2-(2-(2-(2-(4-((*E*)-16-(9-Ethyl-9*H*-carbazol-3-yl)-14-(4-iodobenzoyl)-2,5,8,11-tetraoxa-15-azahexadec-15-en-1-yl)-1*H*-1,2,3-triazol-1-yl)ethoxy)ethoxy)ethoxy)ethyl)-5-((3*aS*,4*S*,6*aR*)-2-oxohexahydro-1*H*-thieno[3,4-*d*]imidazol-4-yl)pentanamide (S11) (*N*-biotinylated NP-14)**

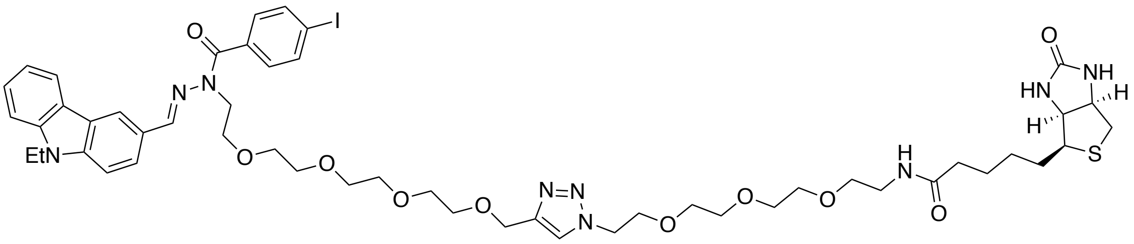

^{1}H NMR (500 MHz, $CDCl_3$) δ 8.27 (s, 1H), 8.18 (d, *J* = 1.1 Hz, 1H), 8.05 (d, *J* = 7.7 Hz, 1H), 7.86–7.76 (m, 2H), 7.71 (s, 1H), 7.66 (dd, *J* = 1.5, 8.6 Hz, 1H), 7.59–7.52 (m, 2H), 7.49 (ddd, *J* = 1.1, 6.1, 8.2 Hz, 1H), 7.42 (d, *J* = 8.2 Hz, 1H), 7.38 (d, *J* = 8.6 Hz, 1H), 7.27 (ddd, *J* = 0.8, 7.2, 8.0 Hz, 1H), 6.63 (t, *J* = 5.5 Hz, 1H), 6.28 (brs, 1H), 5.40 (brs, 1H), 4.63 (s, 2H), 4.50 (t, *J* = 5.1 Hz, 2H), 4.45 (dd, *J* = 5.1, 7.7 Hz, 1H), 4.39 (t, *J* = 6.1 Hz, 2H), 4.36 (t, *J* = 7.2 Hz, 2H), 4.28 (ddd, *J* = 0.9, 4.7, 7.7 Hz, 1H), 3.85 (t, *J* = 5.5 Hz, 2H), 3.84 (t, *J* = 6.0 Hz, 2H), 3.75–3.67 (m, 2H), 3.67–3.61 (m, 6H), 3.61–3.55 (m, 12H), 3.54 (t, *J* = 5.1 Hz, 2H), 3.47–3.35 (m, 2H), 3.11 (dt, *J* = 4.7, 7.3 Hz, 1H), 2.87 (dd, *J* = 5.0, 12.8 Hz, 1H), 2.71 (d, *J* = 12.8 Hz, 1H), 2.18 (t, *J* = 7.8 Hz, 2H), 1.80–1.57 (m, 4H), 1.43 (t, *J* = 7.2 Hz, 3H), 1.41 (tt, *J* = 7.9 Hz, 2H). ^{13}C NMR (125 MHz, $CDCl_3$) δ 173.16, 169.93, 163.71, 144.85, 142.40, 140.84, 140.32, 136.45, 135.18, 131.71, 126.13, 125.78, 124.29, 123.78, 122.98, 122.75, 120.71, 120.45, 119.48, 108.96, 108.80, 96.73, 70.76, 70.59, 70.59, 70.48, 70.48, 70.43, 70.34, 70.31, 70.03, 69.88, 69.61, 69.40, 69.96, 64.53, 61.71, 60.10, 55.44, 50.11, 41.93, 40.46, 39.09, 37.71, 35.83, 28.11, 28.05, 25.49, 13.80. IR (neat) 2868, 1695, 1645, 1418, 1234, 1092, 1007, 746, 731 cm^{-1}. HRMS (ESI-TOF) *m/z* calcd. for $C_{51}H_{69}IN_9O_{10}S^+$ $[M + H]^+$ 1126.3927, found 1126.3942. $[\alpha]^{28}_D$ +13.0 (*c* 1.05, $CHCl_3$).

Synthesis of NP-10 derivatives

The synthesis of NP-10 derivatives was performed according to a reported procedure (1, 4) starting from the corresponding aldehydes and hydrazides, and the structure of the product was identified by analytical methods as reported previously. Some of the derivatives (HMI83-1, HMI83-2, HMI83-3) have been reported (4), and the detailed information for other derivatives is available upon request.

Supplementary References

1. Ohira, M.; Iwasaki, Y.; Tanaka, C.; Kuroki, M.; Matsuo, N.; Kitamura, T.; Yukuhiro, M.; Morimoto, H.; Pang, N.; Liu, B.; Kiyono, T.; Amemiya, M.; Tanaka, K.; Yoshida, K.; Sugimoto, N.; Ohshima, T.; Fujita, M. *Biochim. Biophys. Acta - General Subjects* **2015**, *1850*, 1676.

2. Dao, K.-L.; Sawant, R. R.; Hendricks, J. A.; Ronga, V.; Torchilin, V. P.; Hanson, R. N. *Bioconjugate Chem*. **2012**, *23*, 785.

3. Goswami, L. N.; Houston, Z. H.; Sarma, S. J.; Jalisatgi, S. S.; Hawthorne, M. F. *Org. Biomol. Chem.* **2013**, *11*, 1116.

4. Fujita, M.; Ohshima, T.; Morimoto, H. Benzohydrazide derivative for inducing G2/M phase arrest and cell death. WO 2013/061669 A1, **2013**.

Accession Number	Score for Active NP-10	Score for Inactive NP-10	The ratio	Protein Name	Classification	MW
Q9NU22	210.1	0.0		Midasin OS=Homo sapiens GN=MDN1 PE=1 SV=2 - [MDN1_HUMAN]	Other	632.4
P50748	768.9	0.0		Kinetochore-associated protein 1 OS=Homo sapiens GN=KNTC1 PE=1 SV=1 - [KNTC1_HUMAN]	Mitosis	250.6
Q13395	687.6	0.0		Probable methyltransferase TARBP1 OS=Homo sapiens GN=TARBP1 PE=1 SV=1 - [TARB1_HUMAN]	Other	181.6
Q7Z3U7	563.1	0.0		Protein MON2 homolog OS=Homo sapiens GN=MON2 PE=1 SV=3 - [MON2_HUMAN]	Vesicle-mediated transport	190.2
P07814	550.0	0.0		Bifunctional glutamate/proline--tRNA ligase OS=Homo sapiens GN=EPRS PE=1 SV=5 - [SYEP_HUMAN]	Other	170.5
Q5VYK3	549.6	0.0		Proteasome-associated protein ECM29 homolog OS=Homo sapiens GN=ECM29 PE=1 SV=2 - [ECM29_HUMAN]	Other	204.2
Q15021	547.8	0.0		Condensin complex subunit 1 OS=Homo sapiens GN=NCAPD2 PE=1 SV=3 - [CND1_HUMAN]	Mitosis	157.1
P41252	478.0	0.0		Isoleucine--tRNA ligase, cytoplasmic OS=Homo sapiens GN=IARS PE=1 SV=2 - [SYIC_HUMAN]	Other	144.4
Q6R327	474.2	0.0		Rapamycin-insensitive companion of mTOR OS=Homo sapiens GN=RICTOR PE=1 SV=1 - [RICTR_HUMAN]	Cytoskeletal regulation	192.1
Q6P2E9	470.8	0.0		Enhancer of mRNA-decapping protein 4 OS=Homo sapiens GN=EDC4 PE=1 SV=1 - [EDC4_HUMAN]	Other	151.6
Q9Y6D5	456.4	0.0		Brefeldin A-inhibited guanine nucleotide-exchange protein 2 OS=Homo sapiens GN=ARFGEF2 PE=1 SV=3 - [BIG2_HUMAN]	Vesicle-mediated transport	201.9
Q9NVI1	409.6	0.0		Fanconi anemia group I protein OS=Homo sapiens GN=FANCI PE=1 SV=4 - [FANCI_HUMAN]	Other	149.2
O75533	353.1	0.0		Splicing factor 3B subunit 1 OS=Homo sapiens GN=SF3B1 PE=1 SV=3 - [SF3B1_HUMAN]	Other	145.7
P46379	339.2	0.0		Large proline-rich protein BAG6 OS=Homo sapiens GN=BAG6 PE=1 SV=2 - [BAG6_HUMAN]	Other	119.3
O75448	317.9	0.0		Mediator of RNA polymerase II transcription subunit 24 OS=Homo sapiens GN=MED24 PE=1 SV=1 - [MED24_HUMAN]	Other	110.2
Q92621	316.4	0.0		Nuclear pore complex protein Nup205 OS=Homo sapiens GN=NUP205 PE=1 SV=3 - [NU205_HUMAN]	Nuclear pore organization	227.8
Q5T4S7	314.0	0.0		E3 ubiquitin-protein ligase UBR4 OS=Homo sapiens GN=UBR4 PE=1 SV=1 - [UBR4_HUMAN]	Other	573.5
Q27J81	285.9	0.0		Inverted formin-2 OS=Homo sapiens GN=INF2 PE=1 SV=2 - [INF2_HUMAN]	Cytoskeletal regulation	135.5
Q8NDA8	281.6	0.0		Maestro heat-like repeat-containing protein family member 1 OS=Homo sapiens GN=MROH1 PE=2 SV=3 - [MROH1_HUMAN]	Other	181.1
Q8WTW3	238.1	0.0		Conserved oligomeric Golgi complex subunit 1 OS=Homo sapiens GN=COG1 PE=1 SV=1 - [COG1_HUMAN]	Vesicle-mediated transport	108.9
Q5SRE5	235.2	0.0		Nucleoporin NUP188 homolog OS=Homo sapiens GN=NUP188 PE=1 SV=1 - [NU188_HUMAN]	Nuclear pore organization	195.9
Q14746	230.3	0.0		Conserved oligomeric Golgi complex subunit 2 OS=Homo sapiens GN=COG2 PE=1 SV=1 - [COG2_HUMAN]	Vesicle-mediated transport	83.2
Q9NSG2	226.0	0.0		Uncharacterized protein C1orf112 OS=Homo sapiens GN=C1orf112 PE=1 SV=1 - [CA112_HUMAN]	Other	96.5
P53621	202.2	0.0		Coatomer subunit alpha OS=Homo sapiens GN=COPA PE=1 SV=2 - [COPA_HUMAN]	Vesicle-mediated transport	138.3
Q92538	201.2	0.0		Golgi-specific brefeldin A-resistance guanine nucleotide exchange factor 1 OS=Homo sapiens GN=GBF1 PE=1 SV=2 - [GBF1_HUMAN]	Vesicle-mediated transport	206.3
Q9H0U4	365.8	0.0		Ras-related protein Rab-1B OS=Homo sapiens GN=RAB1B PE=1 SV=1 - [RAB1B_HUMAN]	Vesicle-mediated transport	22.2
P61224	321.4	0.0		Ras-related protein Rap-1b OS=Homo sapiens GN=RAP1B PE=1 SV=1 - [RAP1B_HUMAN]	Other	20.8
P12236	281.5	0.0		ADP/ATP translocase 3 OS=Homo sapiens GN=SLC25A6 PE=1 SV=4 - [ADT3_HUMAN]	Other	32.8
P61006	277.2	0.0		Ras-related protein Rab-8A OS=Homo sapiens GN=RAB8A PE=1 SV=1 - [RAB8A_HUMAN]	Vesicle-mediated transport	23.7
Q9HAV4	949.3	152.2	6.237	Exportin-5 OS=Homo sapiens GN=XPO5 PE=1 SV=1 - [XPO5_HUMAN]	Nuclear transport	136.2
P42345	830.3	166.8	4.977	Serine/threonine-protein kinase mTOR OS=Homo sapiens GN=MTOR PE=1 SV=1 - [MTOR_HUMAN]	Other	288.7
O75694	820.9	177.1	4.636	Nuclear pore complex protein Nup155 OS=Homo sapiens GN=NUP155 PE=1 SV=1 - [NU155_HUMAN]	Nuclear pore organization	155.1
O95373	2820.3	621.9	4.535	Importin-7 OS=Homo sapiens GN=IPO7 PE=1 SV=1 - [IPO7_HUMAN]	Nuclear transport	119.4
O43156	641.3	144.3	4.444	TELO2-interacting protein 1 homolog OS=Homo sapiens GN=TTI1 PE=1 SV=3 - [TTI1_HUMAN]	Other	122.0
P35579	488.3	111.7	4.372	Myosin-9 OS=Homo sapiens GN=MYH9 PE=1 SV=4 - [MYH9_HUMAN]	Cytoskeletal regulation	226.4
P21333	716.9	194.1	3.694	Filamin-A OS=Homo sapiens GN=FLNA PE=1 SV=4 - [FLNA_HUMAN]	Cytoskeletal regulation	280.6
Q96QU8	463.6	150.8	3.074	Exportin-6 OS=Homo sapiens GN=XPO6 PE=1 SV=1 - [XPO6_HUMAN]	Nuclear transport	128.8
Q14974	1246.0	410.3	3.037	Importin subunit beta-1 OS=Homo sapiens GN=KPNB1 PE=1 SV=2 - [IMB1_HUMAN]	Nuclear transport	97.1
P27708	633.7	215.6	2.939	CAD protein OS=Homo sapiens GN=CAD PE=1 SV=3 - [PYR1_HUMAN]	Other	242.8
Q00610	1722.1	599.6	2.872	Clathrin heavy chain 1 OS=Homo sapiens GN=CLTC PE=1 SV=5 - [CLH1_HUMAN]	Mitosis	191.5
Q9Y2V7	275.0	96.1	2.861	Conserved oligomeric Golgi complex subunit 6 OS=Homo sapiens GN=COG6 PE=1 SV=2 - [COG6_HUMAN]	Vesicle-mediated transport	73.2
P57678	613.0	218.9	2.800	Gem-associated protein 4 OS=Homo sapiens GN=GEMIN4 PE=1 SV=2 - [GEMI4_HUMAN]	Other	120.0
P15880	264.3	95.0	2.783	40S ribosomal protein S2 OS=Homo sapiens GN=RPS2 PE=1 SV=2 - [RS2_HUMAN]	Other	31.3
Q9C0E2	324.6	117.3	2.767	Exportin-4 OS=Homo sapiens GN=XPO4 PE=1 SV=2 - [XPO4_HUMAN]	Nuclear transport	130.1
Q01082	217.7	79.4	2.741	Spectrin beta chain, non-erythrocytic 1 OS=Homo sapiens GN=SPTBN1 PE=1 SV=2 - [SPTB2_HUMAN]	Cytoskeletal regulation	274.4
Q9UBB4	349.4	134.9	2.590	Ataxin-10 OS=Homo sapiens GN=ATXN10 PE=1 SV=1 - [ATX10_HUMAN]	Other	53.5
O15397	524.7	202.8	2.587	Importin-8 OS=Homo sapiens GN=IPO8 PE=1 SV=2 - [IPO8_HUMAN]	Nuclear transport	119.9
P47897	238.3	94.7	2.517	Glutamine--tRNA ligase OS=Homo sapiens GN=QARS PE=1 SV=1 - [SYQ_HUMAN]	Other	87.7
Q9H900	512.7	222.3	2.307	Protein zwilch homolog OS=Homo sapiens GN=ZWILCH PE=1 SV=2 - [ZWILC_HUMAN]	Mitosis	67.2
P23396	226.3	98.2	2.305	40S ribosomal protein S3 OS=Homo sapiens GN=RPS3 PE=1 SV=2 - [RS3_HUMAN]	Other	26.7
P62805	276.0	122.1	2.261	Histone H4 OS=Homo sapiens GN=HIST1H4A PE=1 SV=2 - [H4_HUMAN]	Other	11.4
P57740	252.8	112.3	2.251	Nuclear pore complex protein Nup107 OS=Homo sapiens GN=NUP107 PE=1 SV=1 - [NU107_HUMAN]	Nuclear pore organization	106.3
Q9UP83	249.0	121.5	2.050	Conserved oligomeric Golgi complex subunit 5 OS=Homo sapiens GN=COG5 PE=1 SV=3 - [COG5_HUMAN]	Vesicle-mediated transport	92.7
Q9Y5L0	690.6	340.3	2.030	Transportin-3 OS=Homo sapiens GN=TNPO3 PE=1 SV=3 - [TNPO3_HUMAN]	Nuclear transport	104.1
O00410	1033.8	509.4	2.029	Importin-5 OS=Homo sapiens GN=IPO5 PE=1 SV=4 - [IPO5_HUMAN]	Nuclear transport	123.5
			From F_06			
			From F_08			
			From F_10			
			From F_12			

Supplementary Figure S1. Identification of active NP-10-bound proteins.

Among the proteins identified with active NP-10 beads, the proteins with Mascot scores >200 and ≥2-fold higher than those of the control samples obtained with inactive NP-10 beads are listed. The accession number, Mascot score for active NP-10, Mascot score for inactive NP-10, the ratio between them (active/inactive), protein name, functional classification based on PANTHER (http://pantherdb.org), and molecular weight (MW) are shown.

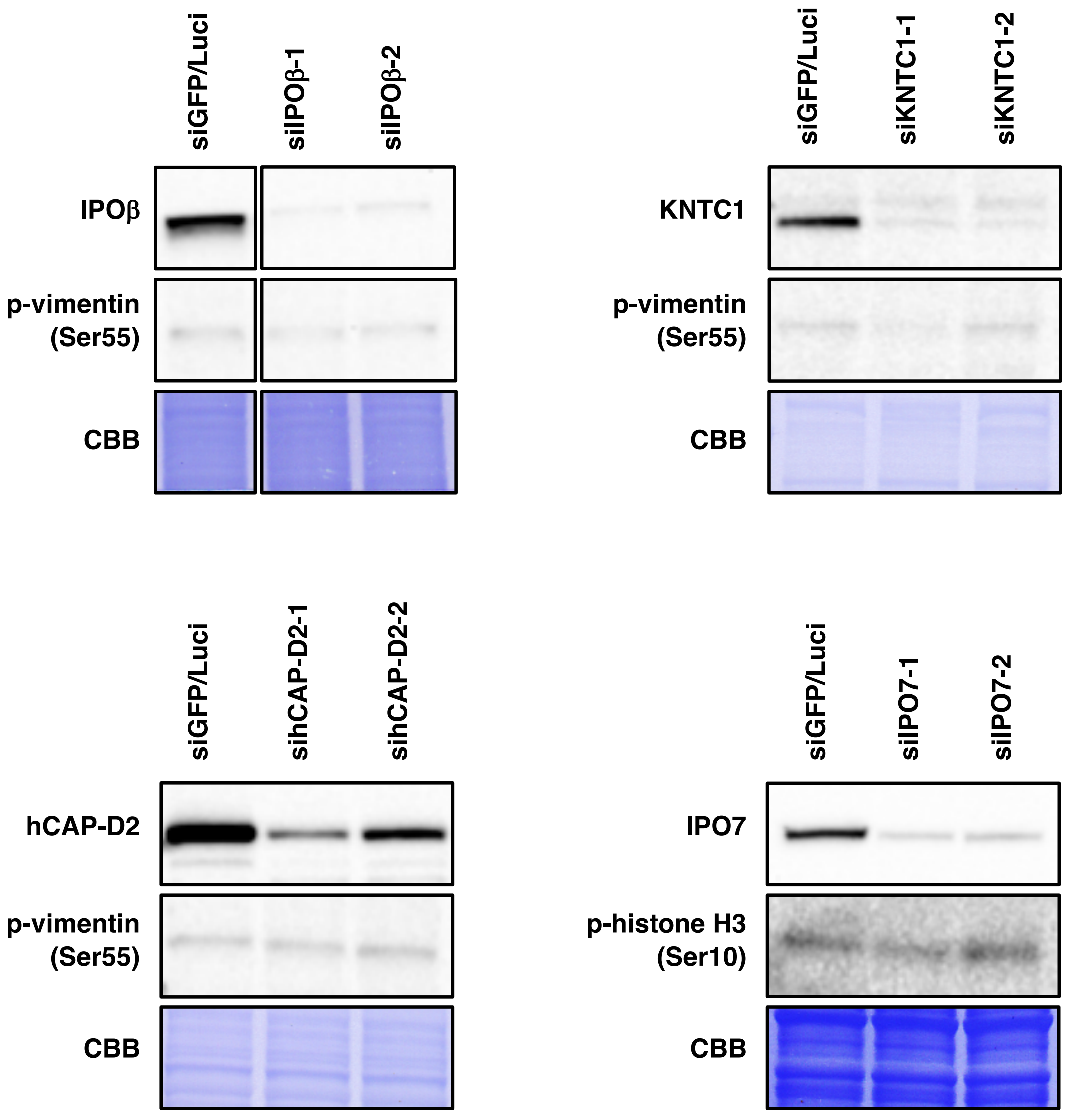

Supplementary Figure S2. Silencing of IPOβ, KNTC1, hCAP, or IPO7, which are active NP-10-bound proteins possibly involved in mitotic regulation, does not induce mitotic arrest.

HeLa cells were transfected with control (mixture of siGFP and siLuci), IPOβ-targeting (siIPOβ-1 or siIPOβ-2), KNTC1-targeting (siKNTC1-1 or siKNTC1-2), hCAP-D2-targeting (sihCAP-D2-1 or sihCAP-D2-2), or IPO7-targeting (siIPO7-1 or siIPO7-2) siRNAs for 48 h. Whole cell extracts were analyzed by immunoblotting with the indicated antibodies. Phospho-vimentin (Ser55), a target of Cdk1 kinase, and phospho-histone H3 (Ser10), a target of Aurora B kinase, were used as mitotic markers.

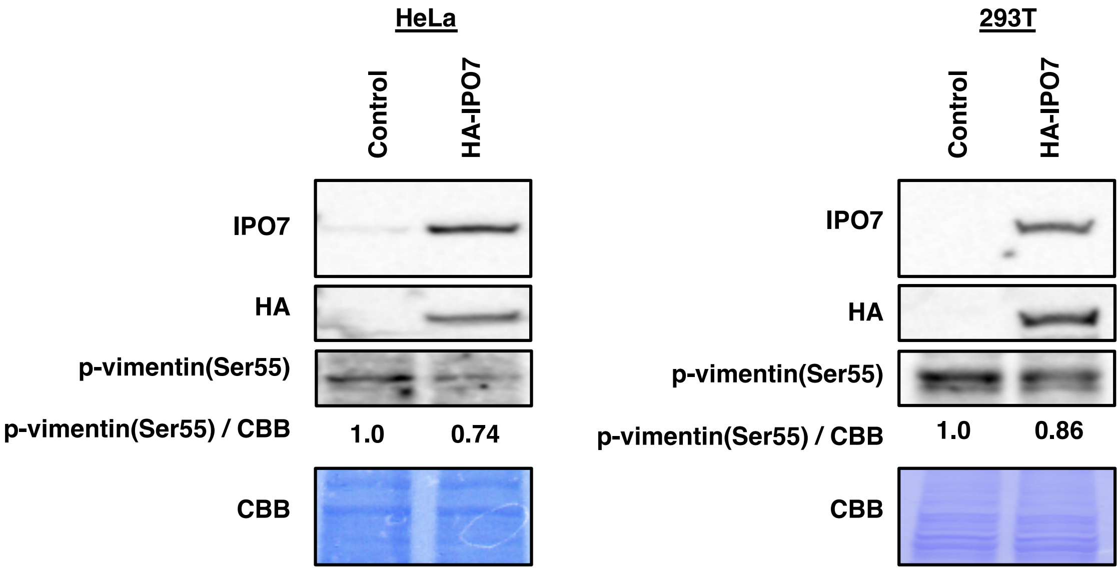

Supplementary Figure S3. Overexpression of IPO7 does not induce mitotic arrest. HeLa and 293T cells were transfected with HA-IPO7 expression vector or empty vector for 48 h and immunoblotted with the indicated antibodies. Phospho-vimentin (Ser55), a target of Cdk1 kinase, was used as mitotic marker. The signal intensities of the bands were quantified and normalized to the signals for CBB bands.

Supplementary Figure S4. **^{1}H NMR analysis of compounds S4, S5, S6, S7, S8, S9, S10, S11, and HMI83-2.**

Current Data Parameters
NAME HMI1342oIPEGLPrecolumn
EXPNO 10
PROCNO 1

F2 - Acquisition Parameters
Date_ 20150116
Time 13.18
INSTRUM spect
PROBHD 5 mm CPPBBO BB
PULPROG zg30
TD 65536
SOLVENT CDCl3
NS 1
DS 0
SWH 8012.820 Hz
FIDRES 0.122266 Hz
AQ 4.0894465 sec
RG 31.29
DW 62.400 usec
DE 10.00 usec
TE 300.2 K
D1 1.00000000 sec
TD0 1

======== CHANNEL f1 ========
SFO1 500.1730010 MHz
NUC1 1H
P1 12.00 usec
PLW1 13.50000000 W

F2 - Processing parameters
SI 65536
SF 500.1700133 MHz
WDW EM
SSB 0
LB 0.30 Hz
GB 0
PC 1.00

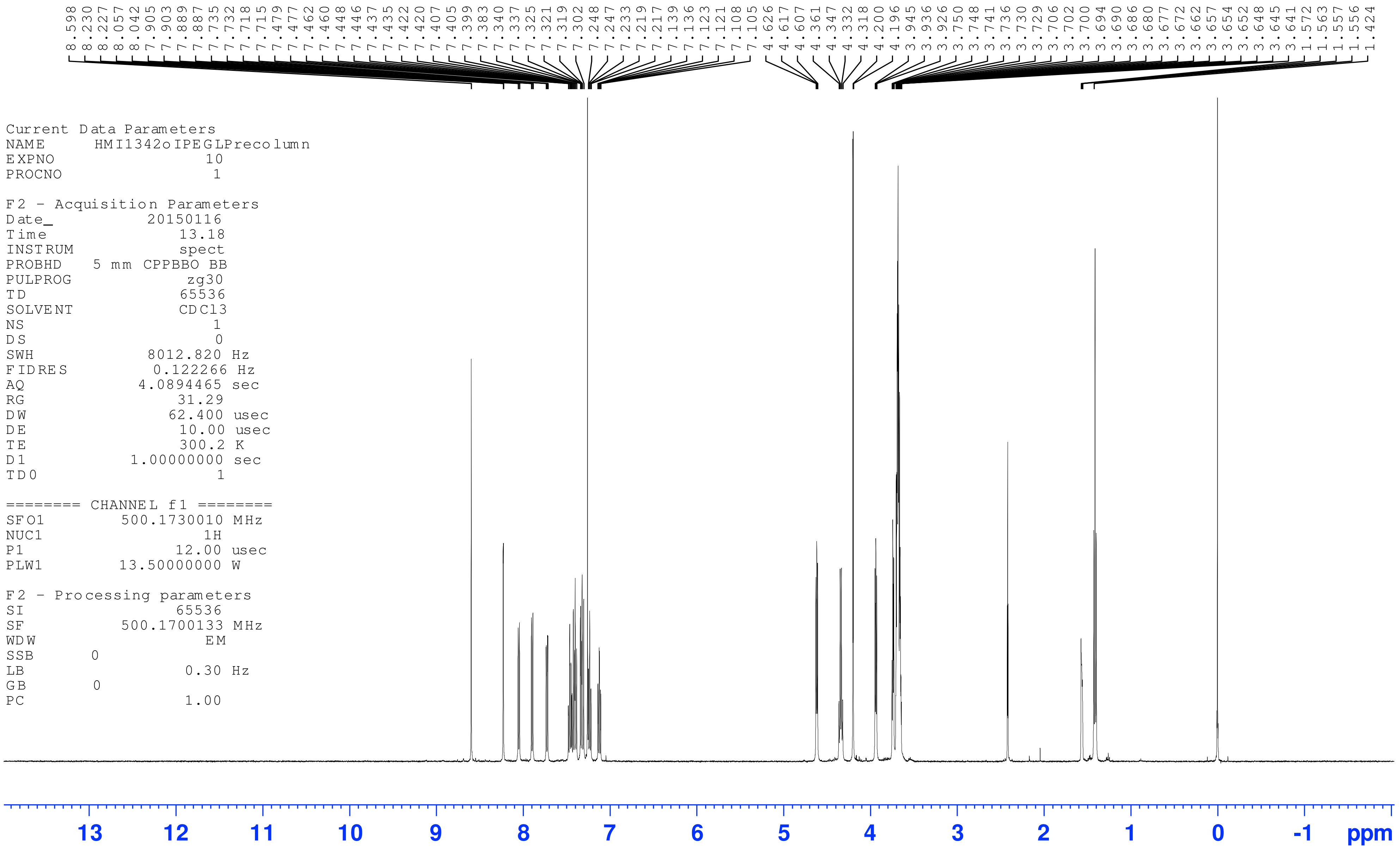

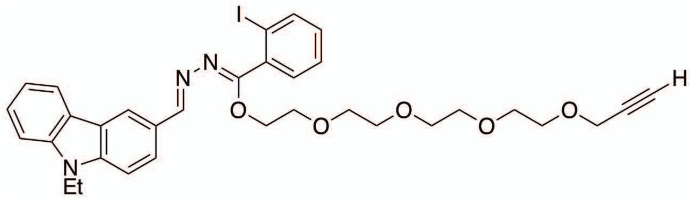

S4 ^{1}H NMR

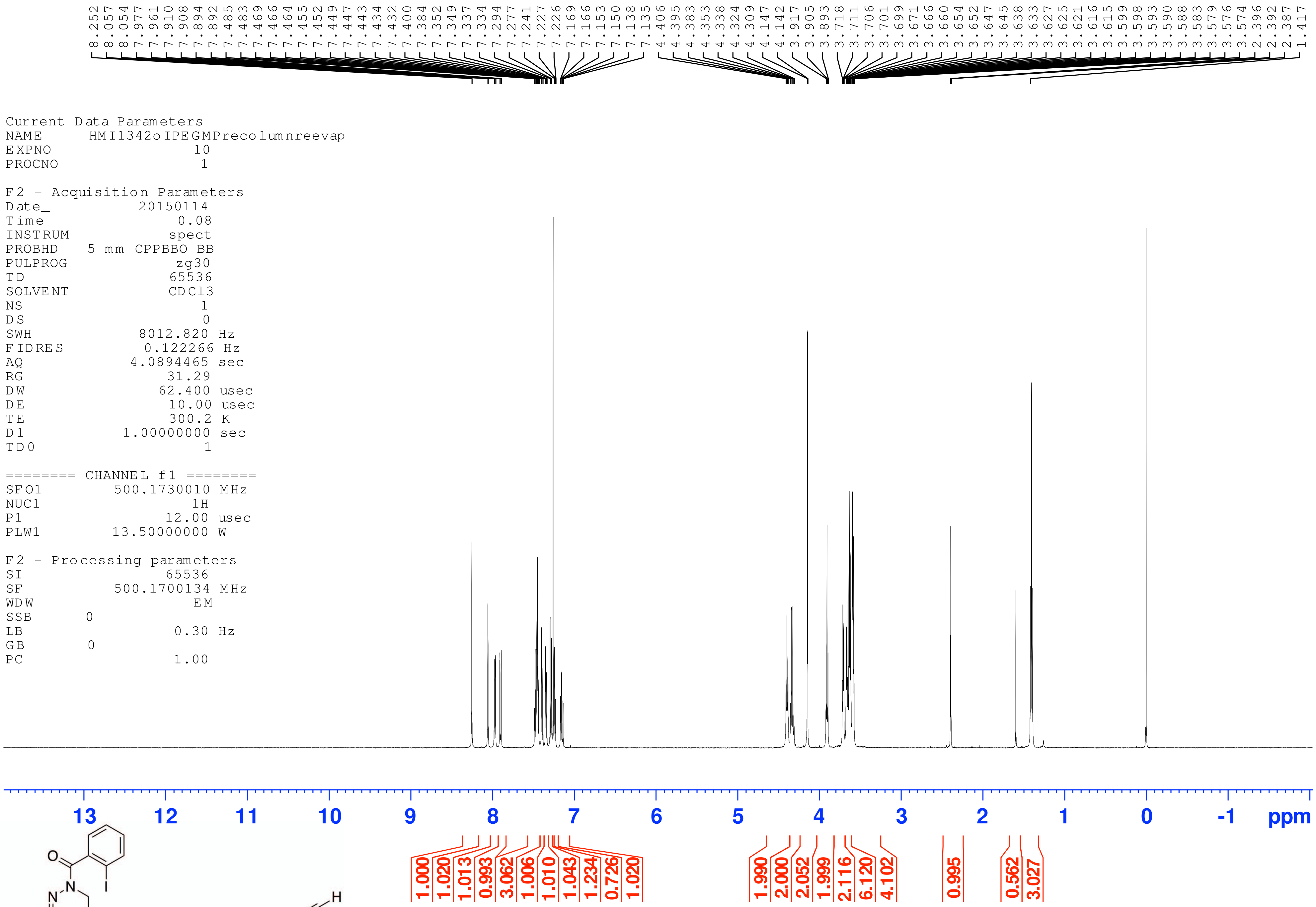
8.252
8.057
8.054
7.977
7.961
7.910
7.908
7.894
7.892
7.485
7.483
7.469
7.466
7.464
7.455
7.452
7.449
7.447
7.443
7.434
7.432
7.400
7.384
7.352
7.349
7.337
7.334
7.294
7.277
7.241
7.227
7.226
7.169
7.166
7.153
7.150
7.138
7.135
4.406
4.395
4.383
4.353
4.338
4.324
4.309
4.147
4.142
3.917
3.905
3.893
3.718
3.711
3.706
3.701
3.699
3.671
3.666
3.660
3.654
3.652
3.647
3.645
3.638
3.633
3.627
3.625
3.621
3.616
3.615
3.599
3.598
3.593
3.590
3.588
3.583
3.579
3.576
3.574
2.396
2.392
2.387
1.417
Current Data Parameters
NAME HMI1342oIPEGMPrecolumnreevap
EXPNO 10
PROCNO 1
F2 - Acquisition Parameters
Date_ 20150114
Time 0.08
INSTRUM spect
PROBHD 5 mm CPPBBO BB
PULPROG zg30
TD 65536
SOLVENT CDCl3
NS 1
DS 0
SWH 8012.820 Hz
FIDRES 0.122266 Hz
AQ 4.0894465 sec
RG 31.29
DW 62.400 usec
DE 10.00 usec
TE 300.2 K
D1 1.00000000 sec
TD0 1
======== CHANNEL f1 ========
SFO1 500.1730010 MHz
NUC1 1H
P1 12.00 usec
PLW1 13.50000000 W
F2 - Processing parameters
SI 65536
SF 500.1700134 MHz
WDW EM
SSB 0
LB 0.30 Hz
GB 0
PC 1.00
13
12
11
10
9
8
7
6
5
4
3
2
1
0
-1
ppm
1.000
1.020
1.013
0.993
3.062
1.006
1.010
1.043
1.234
0.726
1.020
1.990
2.000
2.052
1.999
2.116
6.120
4.102
0.995
0.562
3.027

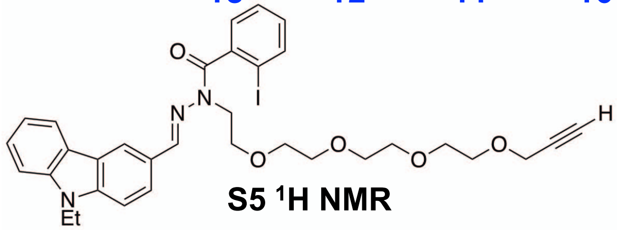
O
N
N
I
O
O
O
O
H
N
Et

S5 ¹H NMR

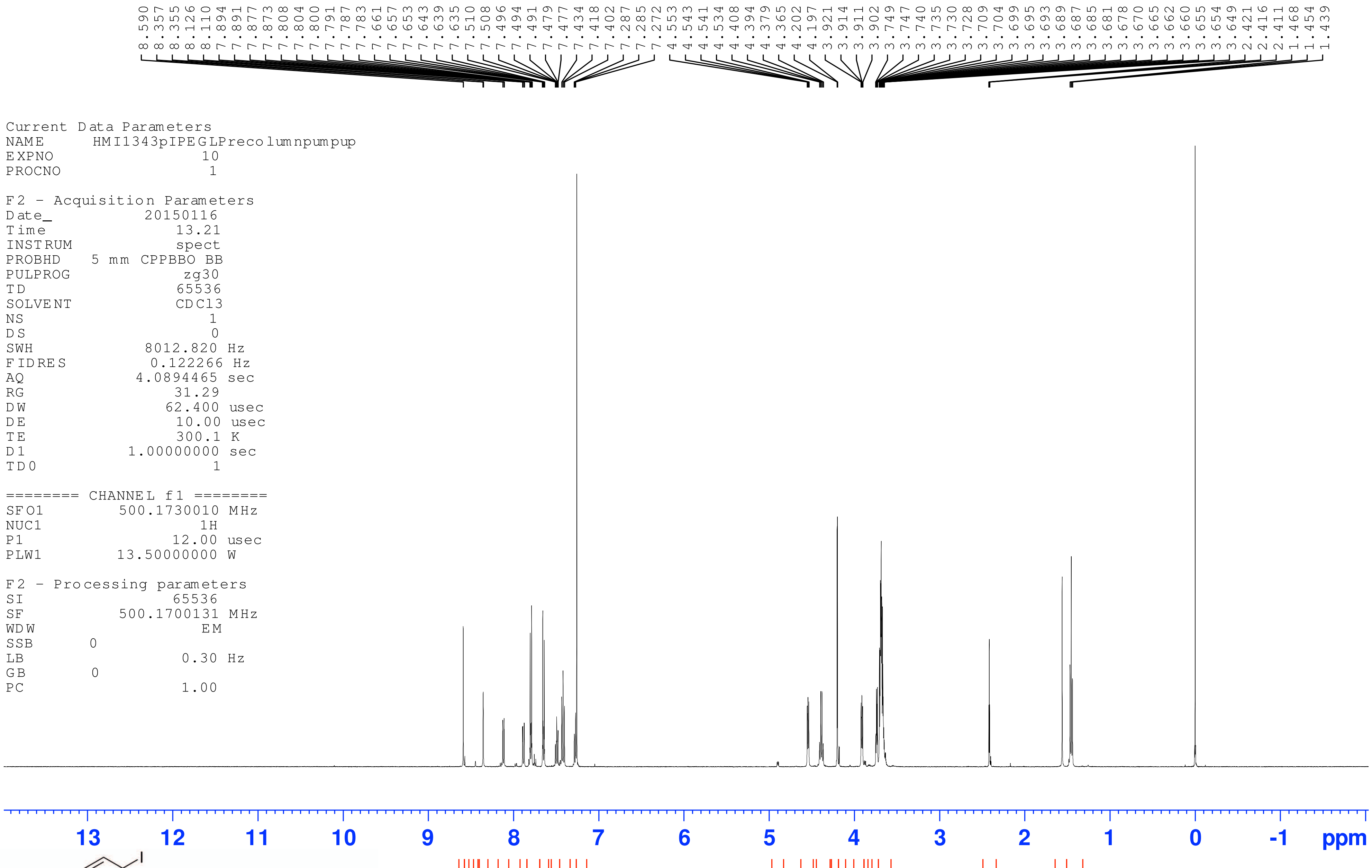
8.590 8.357 8.355 8.126 8.110 7.894 7.891 7.877 7.873 7.808 7.804 7.800 7.791 7.787 7.783 7.661 7.657 7.653 7.643 7.639 7.635 7.510 7.508 7.496 7.494 7.491 7.479 7.477 7.434 7.418 7.402 7.287 7.285 7.272 4.553 4.543 4.541 4.534 4.408 4.394 4.379 4.365 4.202 4.197 3.921 3.914 3.911 3.902 3.749 3.747 3.740 3.735 3.730 3.728 3.709 3.704 3.699 3.695 3.693 3.689 3.687 3.685 3.681 3.678 3.670 3.665 3.662 3.660 3.655 3.654 3.649 2.421 2.416 2.411 1.468 1.454 1.439
Current Data Parameters
NAME HMI1343pIPEGLPrecolumnpumpup
EXPNO 10
PROCNO 1
F2 - Acquisition Parameters
Date_ 20150116
Time 13.21
INSTRUM spect
PROBHD 5 mm CPPBBO BB
PULPROG zg30
TD 65536
SOLVENT CDCl3
NS 1
DS 0
SWH 8012.820 Hz
FIDRES 0.122266 Hz
AQ 4.0894465 sec
RG 31.29
DW 62.400 usec
DE 10.00 usec
TE 300.1 K
D1 1.00000000 sec
TD0 1
======== CHANNEL f1 ========
SFO1 500.1730010 MHz
NUC1 1H
P1 12.00 usec
PLW1 13.50000000 W
F2 - Processing parameters
SI 65536
SF 500.1700131 MHz
WDW EM
SSB 0
LB 0.30 Hz
GB 0
PC 1.00
13 12 11 10 9 8 7 6 5 4 3 2 1 0 -1 ppm
1.000 0.099 0.080 1.017 1.121 1.027 2.442 2.050 1.173 2.232 0.940 2.303 0.147 2.038 2.243 2.048 0.206 2.015 0.199 2.107 11.449 1.088 1.659 3.343

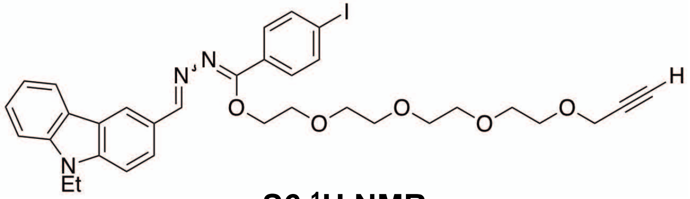
I
N
N
O
O
O
O
O
H
N
Et

S6 ¹H NMR

Current Data Parameters
NAME HMI1343pIPEGMP
EXPNO 10
PROCNO 1

F2 - Acquisition Parameters
Date_ 20150109
Time 0.24
INSTRUM spect
PROBHD 5 mm CPPBBO BB
PULPROG zg30
TD 65536
SOLVENT CDCl3
NS 1
DS 0
SWH 8012.820 Hz
FIDRES 0.122266 Hz
AQ 4.0894465 sec
RG 31.29
DW 62.400 usec
DE 10.00 usec
TE 300.1 K
D1 1.00000000 sec
TD0 1

======== CHANNEL f1 ========
SFO1 500.1730010 MHz
NUC1 1H
P1 12.00 usec
PLW1 13.50000000 W

F2 - Processing parameters
SI 65536
SF 500.1700130 MHz
WDW EM
SSB 0
LB 0.30 Hz
GB 0
PC 1.00

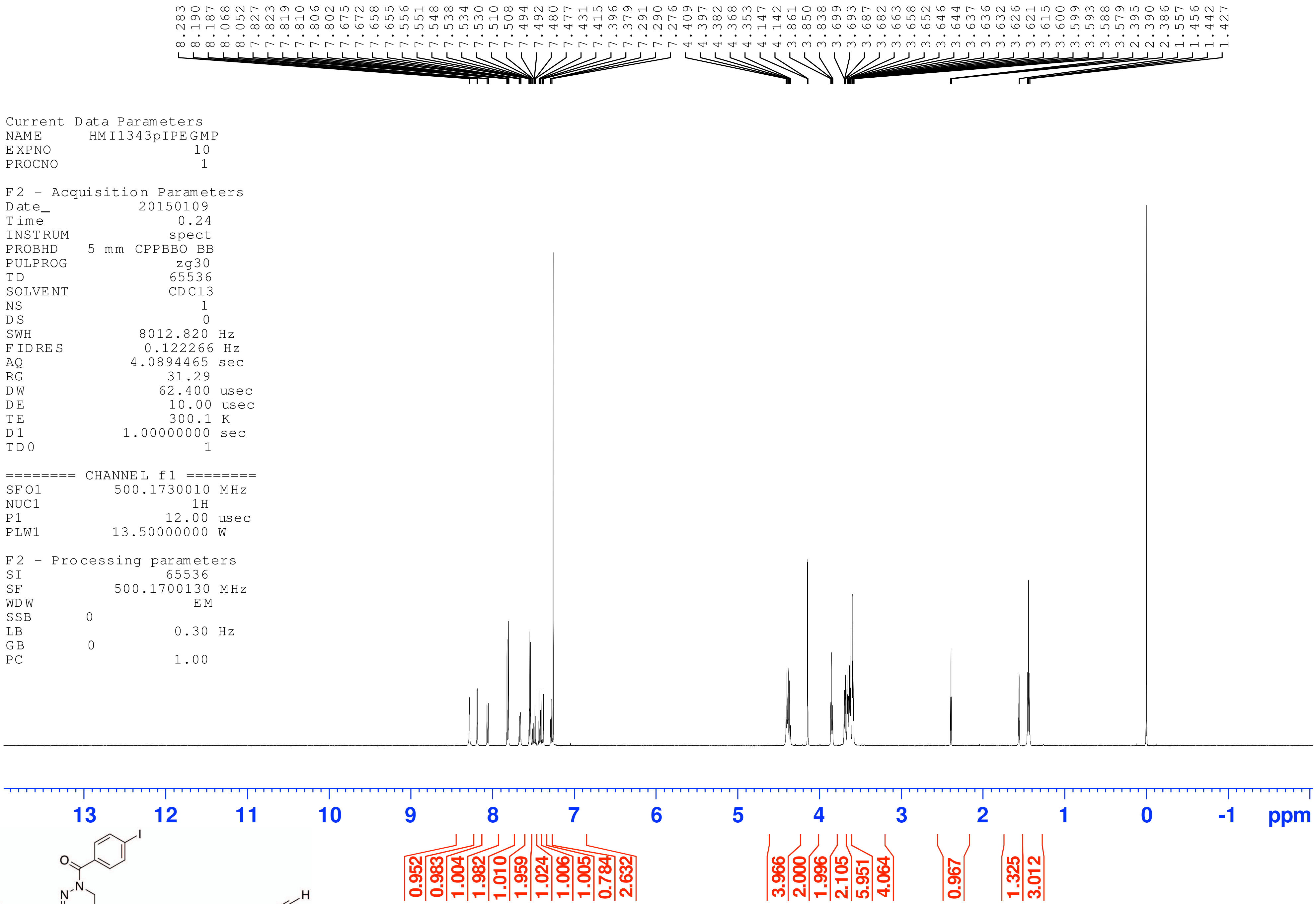

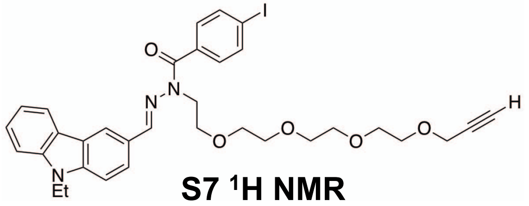

S7 ¹H NMR

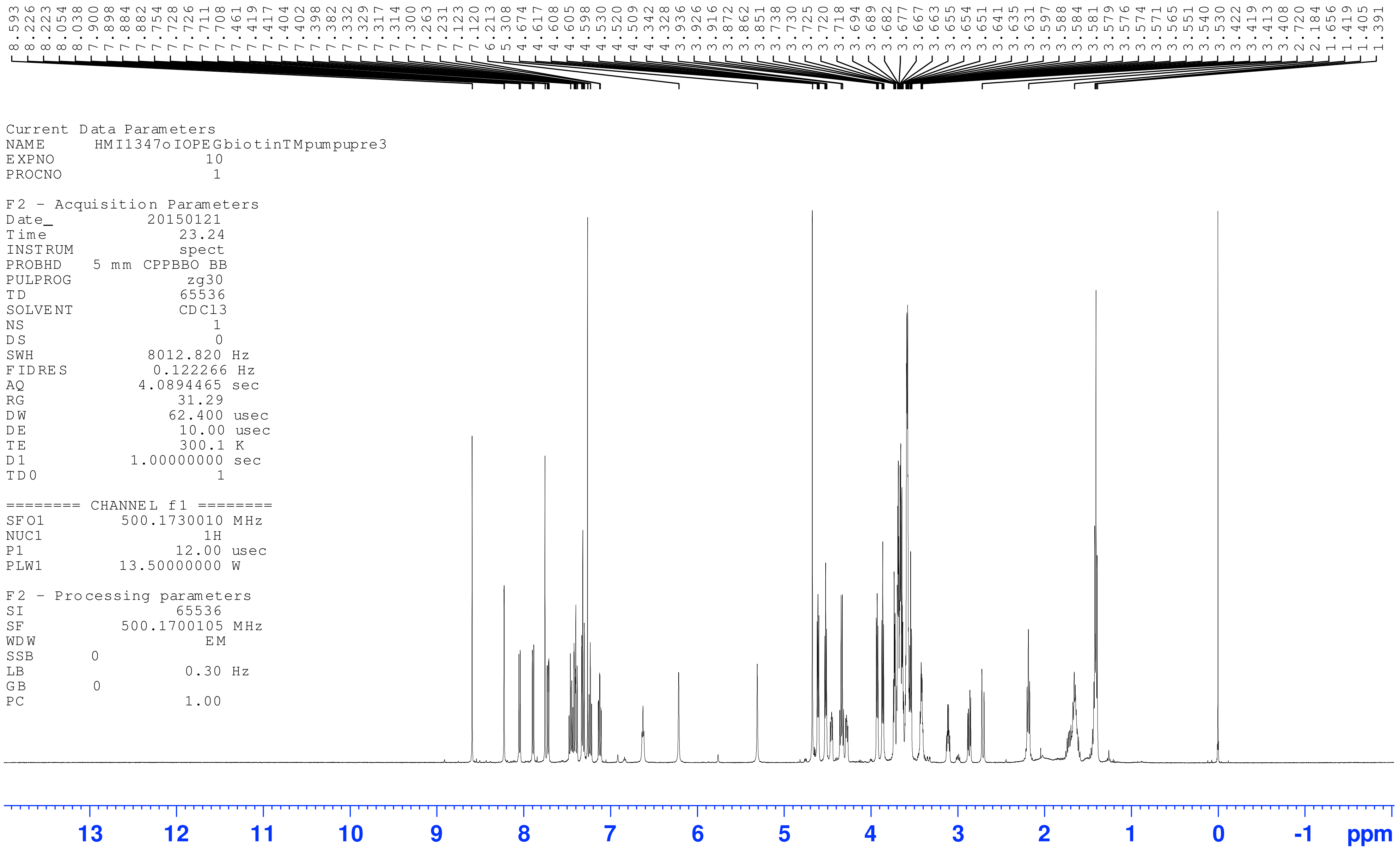

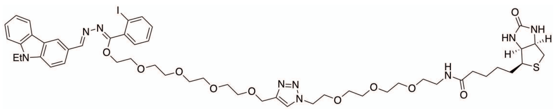

S8 (*O*-biotinylated NP-10) ^{1}H NMR

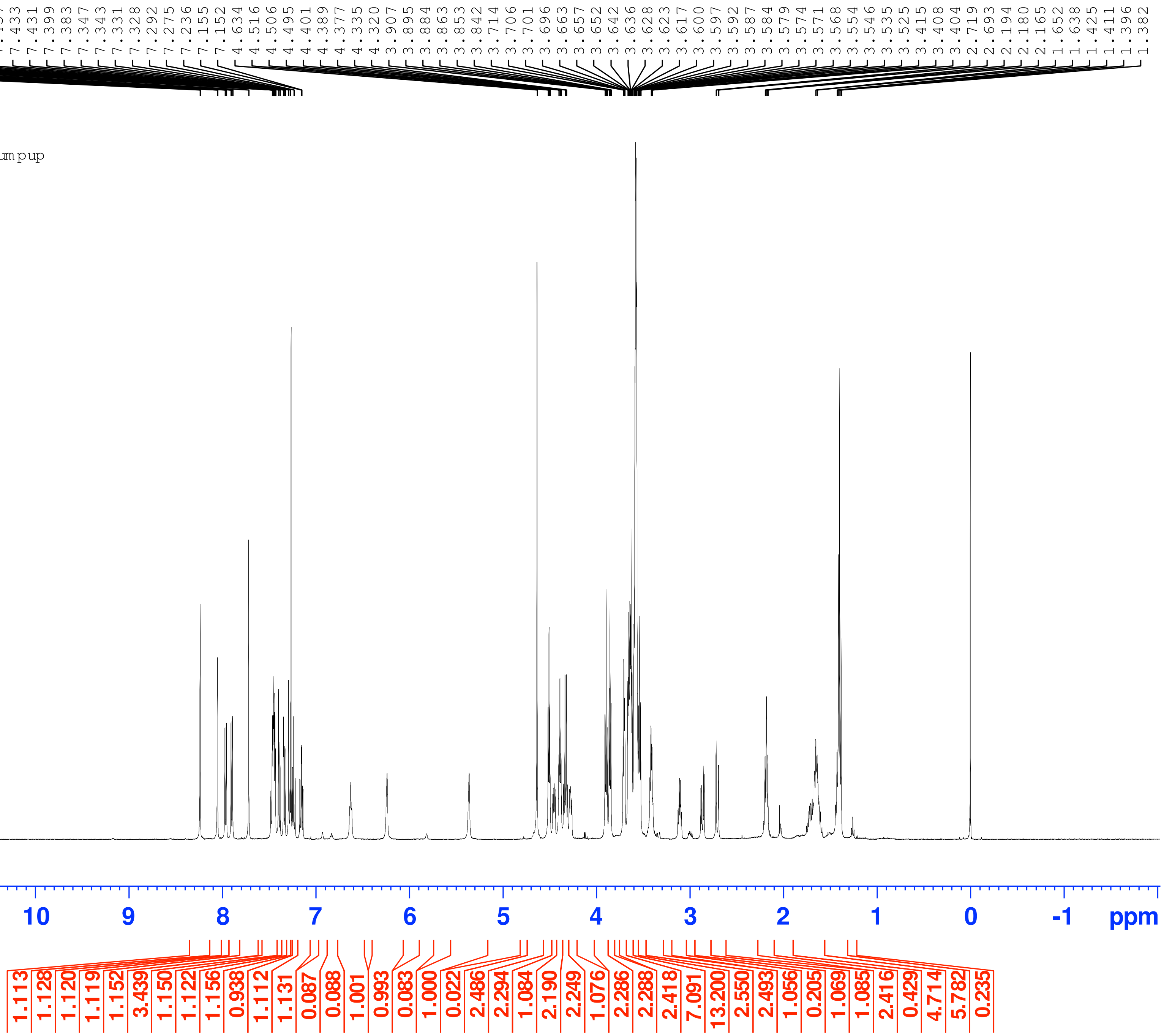

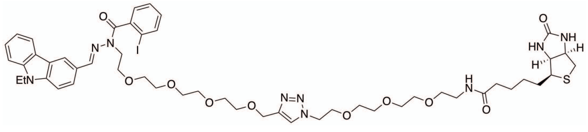

S9 (*N*-biotinylated NP-10) ^{1}H NMR

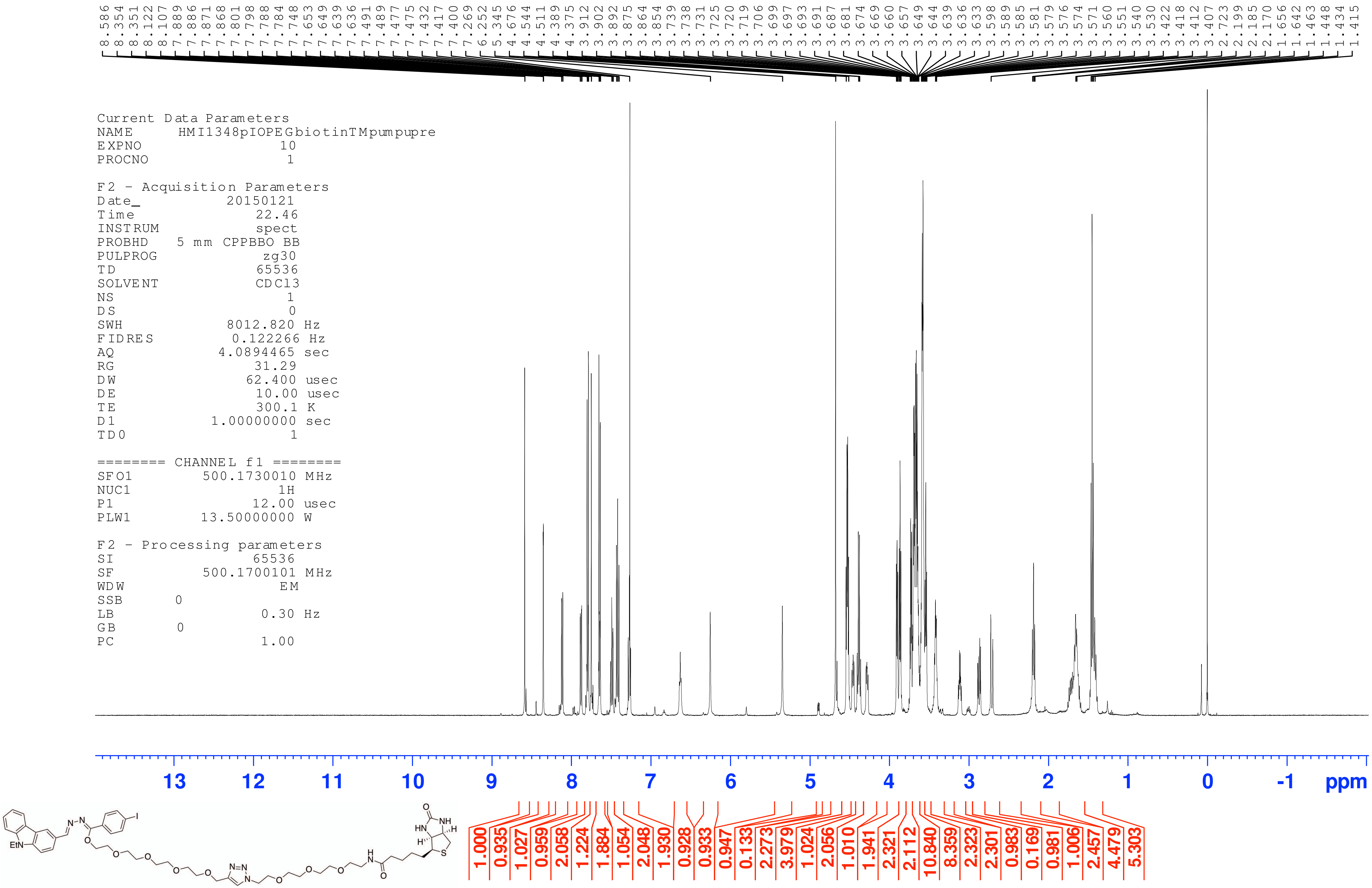

S10 (*O*-biotinylated NP-14) ^{1}H NMR

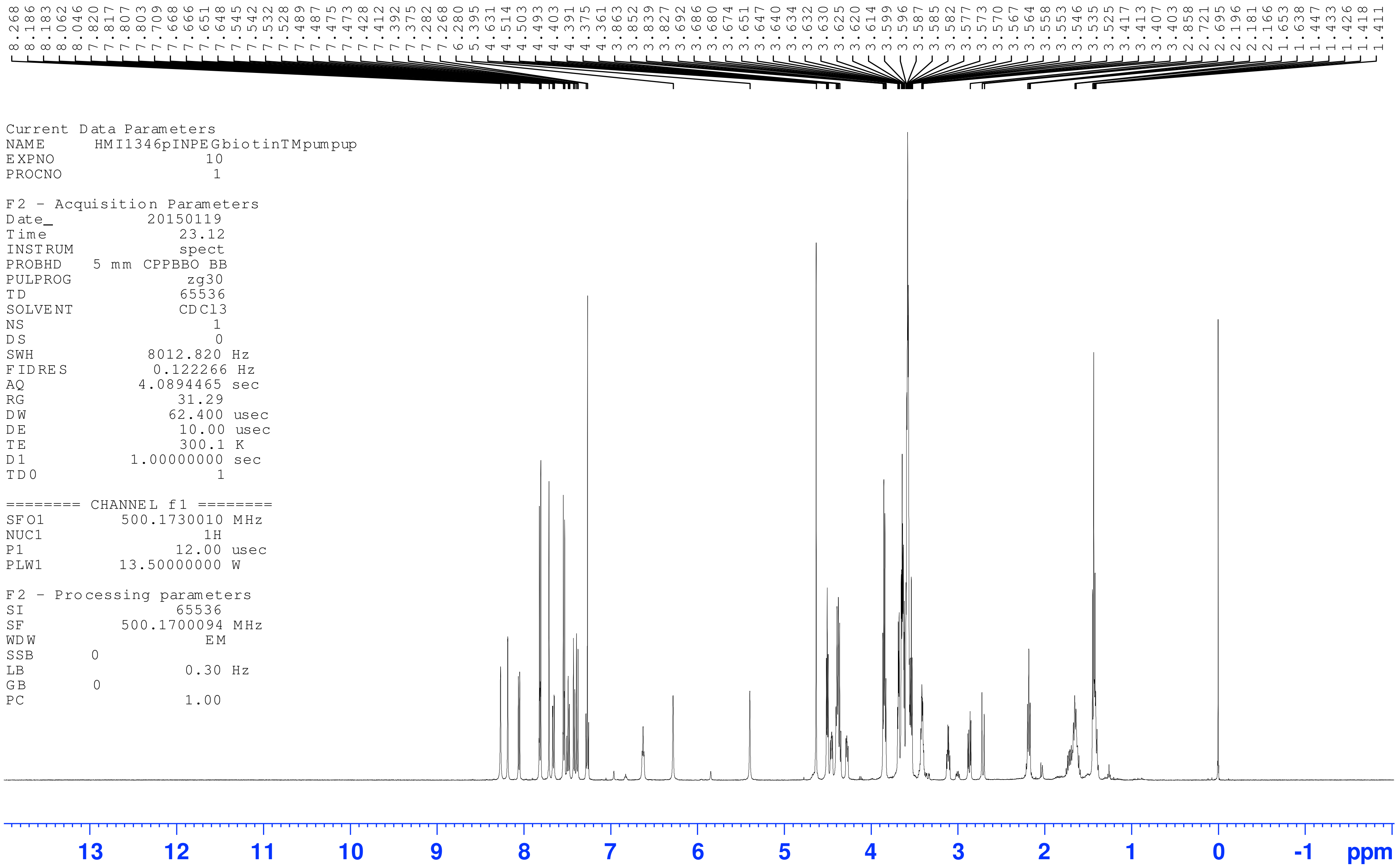

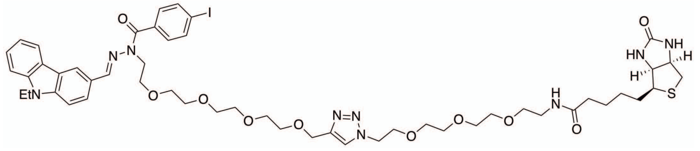

S11 (*N*-biotinylated NP-14) ^{1}H NMR

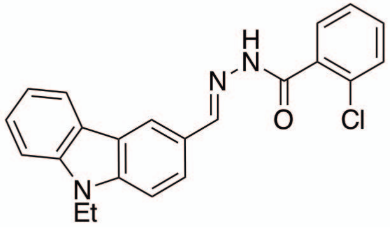

HMI83-2 ¹H NMR

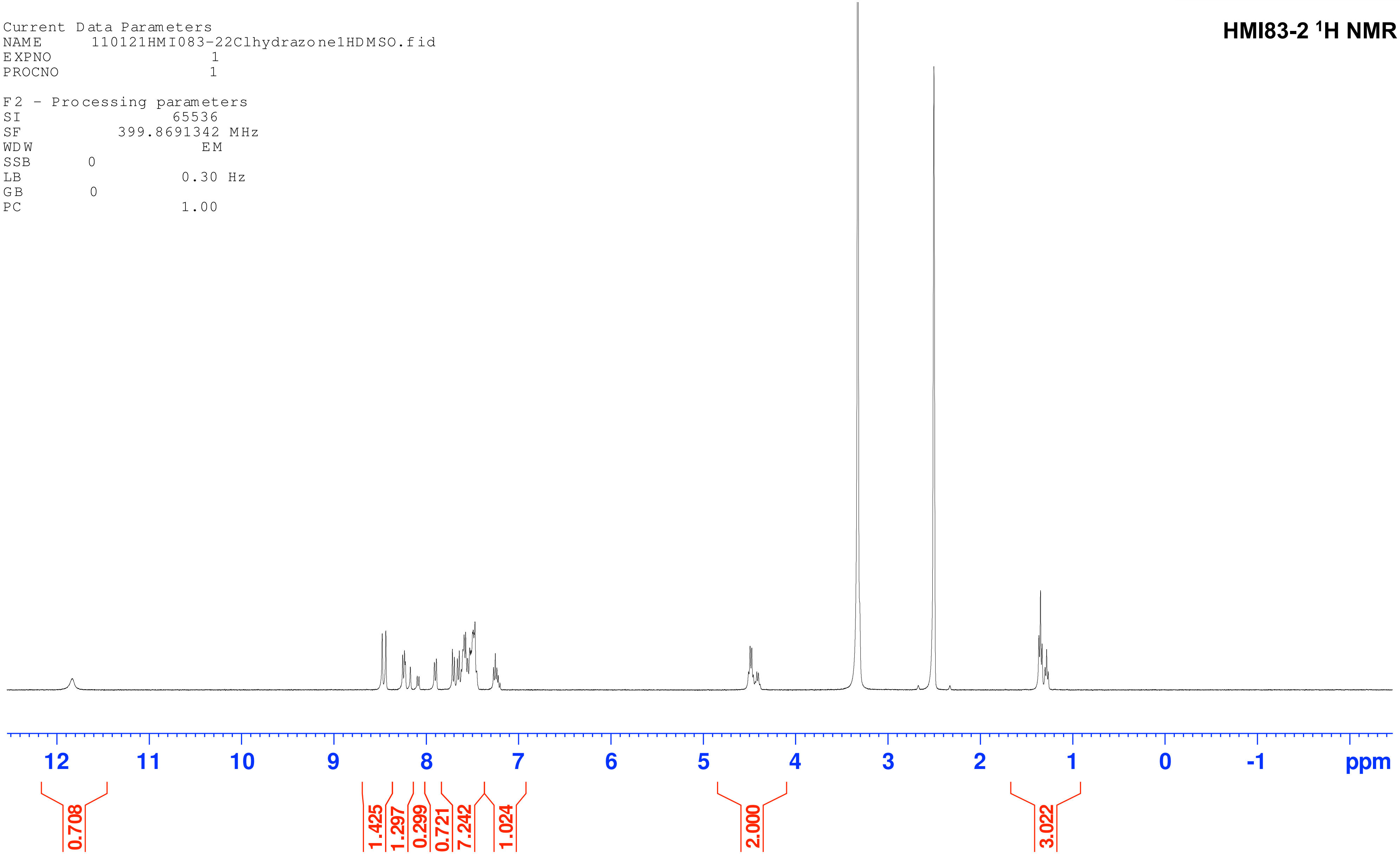

Supplementary Figure S5. Source data for Figures 2, 3, 4, and 5

Source data for Figure 2

Figure 2A. NUP155

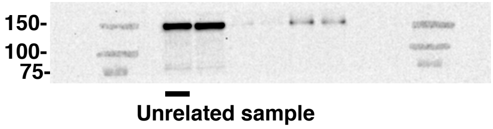

Figure 2A. IPOβ

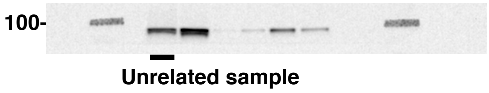

Figure 2A. KNTC1

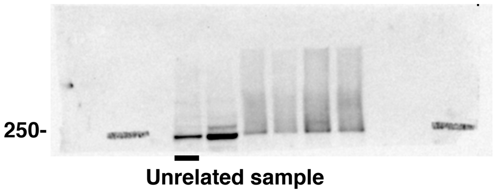

Figure 2A. hCAP-D2

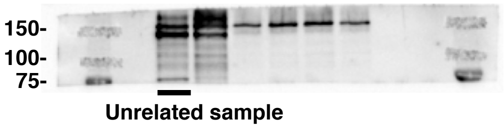

Figure 2A. IPO7

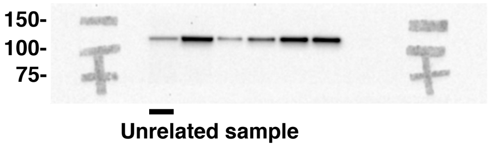

Figure 2B. NUP155

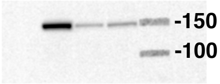

Figure 2B. p–vimentin(Ser55)

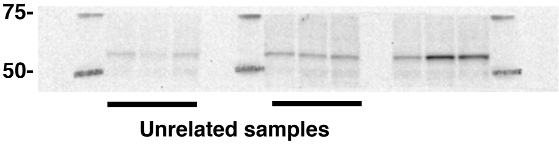

Figure 2B. CBB

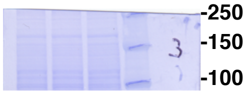

Source data for Figure 3

Figure 3A. NUP155

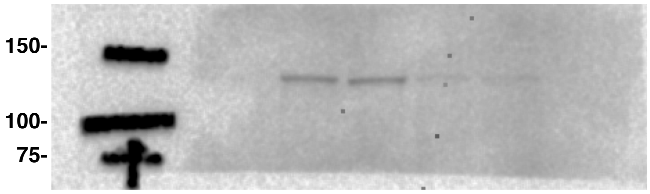

Figure 3A. GFP, IPOβ

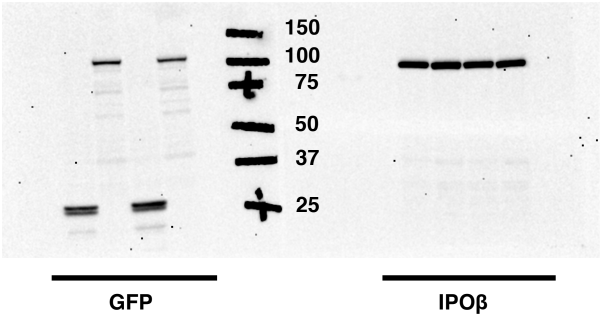

Figure 3A. CBB

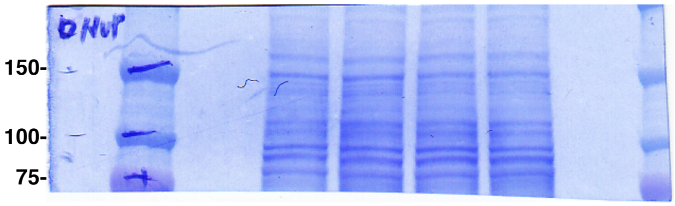

Source data for Figure 4

Figure 4B. GST-NUP155N, GST-NUP155M

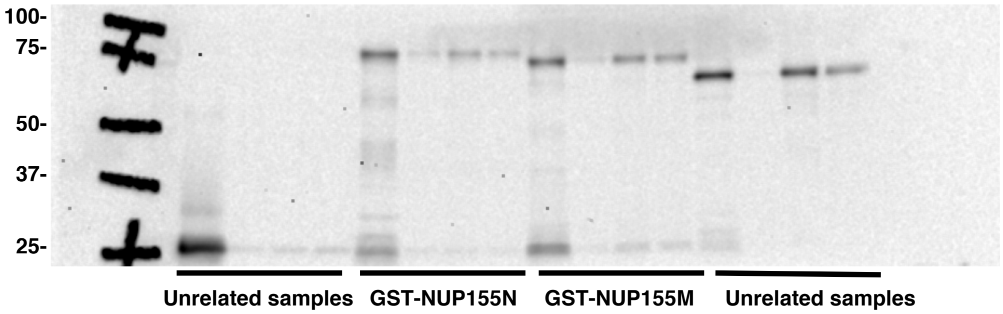

Figure 4B. GST-NUP155C

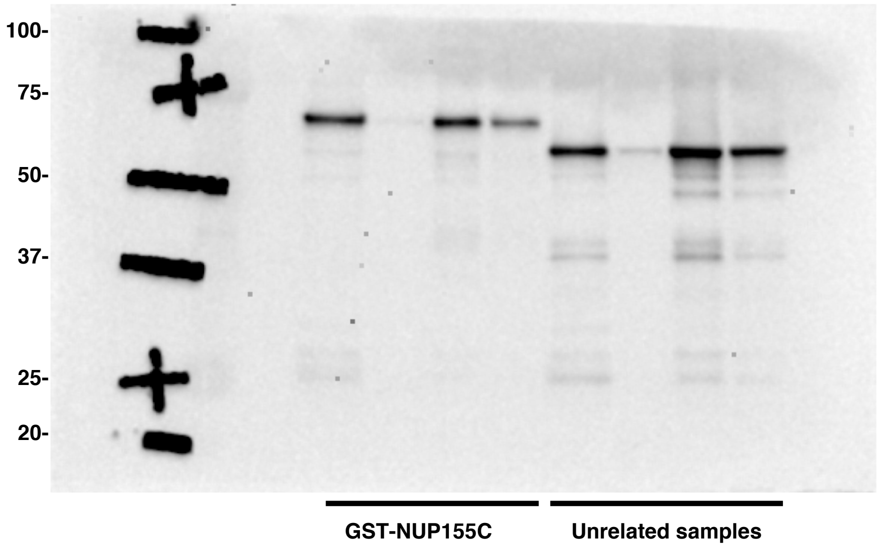

Figure 4B. GST, GST-IPOβN, GST-IPOβC

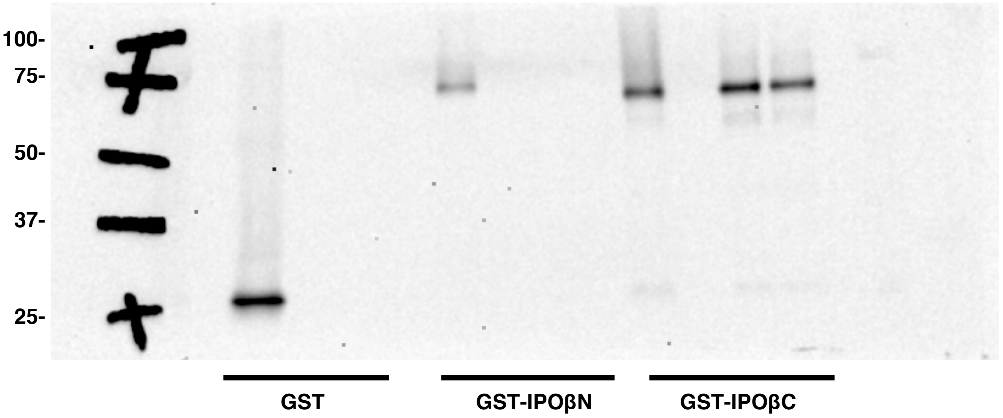

Source data for Figure 5

Figure 5A. GFP-NUP155MC, GFP

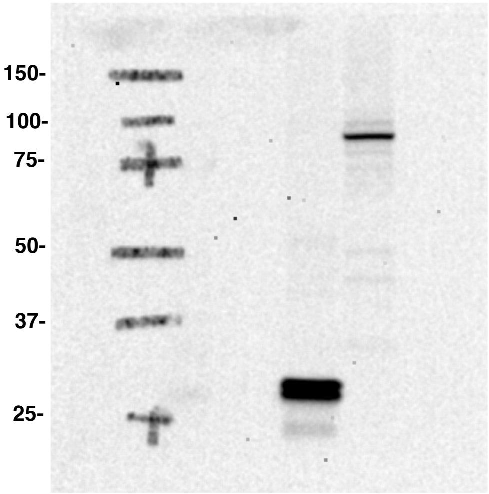

Figure 5A. CBB

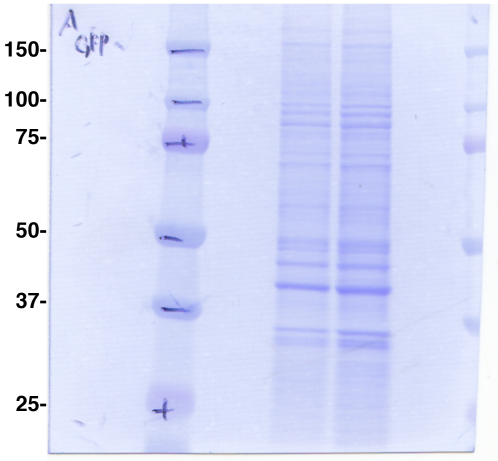

Supplement: Supplementary file 1 — Supplementary information [file 41598_2019_53259_MOESM1_ESM.pdf]
